# Hsa_circ_0004296 inhibits metastasis of prostate cancer by interacting with EIF4A3 to prevent nuclear export of ETS1 mRNA

**DOI:** 10.1186/s13046-021-02138-8

**Published:** 2021-10-25

**Authors:** Shiyu Mao, Wentao Zhang, Fuhan Yang, Yadong Guo, Hong Wang, Yuan Wu, Ruiliang Wang, Niraj Maskey, Zongtai Zheng, Cheng Li, Wenchao Ma, Junfeng Zhang, Yang Yan, Xudong Yao

**Affiliations:** 1 Department of Urology, Shanghai Tenth People’s Hospital, Tongji University School of Medicine, 301 Middle Yan Chang Road, Shanghai, 200072 P. R. China; 2 Shanghai Clinical College, Anhui Medical University, 81 Meishan Road, Hefei, 230032 P. R. China

**Keywords:** Prostate cancer, Metastasis, Hsa_circ_0004296, EIF4A3, ETS1

## Abstract

**Background:**

Circular RNAs (circRNAs) have been shown to play vital biological functions in various tumors, including prostate cancer (PCa). However, the roles of circRNAs in the metastasis of PCa remain unclear. In the present study, differentially expressed circRNAs associated with PCa metastasis were screened using high-throughput RNA sequencing, from which hsa_circ_0004296 was identified.

**Methods:**

Quantitative real-time PCR (qRT-PCR) was used to detect the expression of circ_0004296 in PCa tissues and adjacent normal tissues as well as in blood and urine. Gain and loss of function experiments were performed to investigate the function of circ_0004296 in PCa. Bioinformatics analyses, RNA pull-down assay, and mass spectrometry were conducted to identify RNA-binding proteins. RNA immunoprecipitation and RNA and protein nuclear-cytoplasmic fractionation were performed to investigate the underlying mechanism. A xenograft mouse model was used to analyze the effect of circ_0004296 on PCa growth and metastasis in vivo.

**Results:**

The expression of circ_0004296 was decreased in PCa tissues, blood, and urine, which was negatively associated with metastasis. Furthermore, gain and loss of function experiments in vitro and in vivo showed that circ_0004296 inhibited the proliferation, migration, invasion, and epithelial-mesenchymal transition of PCa cells. Mechanistically, circ_0004296 regulated host gene ETS1 expression at the post-transcriptional level. EIF4A3 was identified and confirmed as the downstream binding protein of circ_0004296. EIF4A3 expression was significantly upregulated in PCa tissues and associated with PCa metastasis. Silencing EIF4A3 suppressed PCa cell proliferation, migration, invasion, and EMT.

**Conclusions:**

Circ_0004296 overexpression efficiently inhibited ETS1 mRNA nuclear export by promoting EIF4A3 retention in the nucleus, leading to the downregulation of ETS1 expression and suppression of PCa metastasis; thus, circ_0004296 might be a potential biomarker and therapeutic target for patients with PCa.

**Supplementary Information:**

The online version contains supplementary material available at 10.1186/s13046-021-02138-8.

## Background

Prostate cancer (PCa) is the second most common malignancy in men and causes around 300,000 deaths worldwide annually [1]. Metastasis is the leading cause of death from PCa [2]. Median survival of patients with newly diagnosed metastasis is approximately 42 months [3], while that of patients with progressive metastatic PCa is approximately 27 months [4]. Therefore, it is important to further clarify the molecular mechanism of PCa metastasis to identify novel biomarkers and develop effective therapeutic targets.

Circular RNAs (circRNAs) are a class of covalently closed circular noncoding RNAs without 5′ and 3′ ends [5]. Although circRNAs were initially considered to be splicing-associated noise, with the advance in high-throughput sequencing methods, a large number of circRNA candidates were found to have potential biological functions. Accumulated evidence has confirmed abnormal expression of circRNAs in multiple tumors, including PCa, wherein they function as oncogenes or tumor suppressor genes [6]. CircRNAs are involved in the malignant behavior of tumor cells, such as proliferation, apoptosis, migration, and invasion [7–9]. At the molecular level, circRNAs play potentially important roles in tumorigenesis and tumor progression through competitive endogenous RNAs (ceRNAs), encoded proteins, interaction with proteins, and modulation of transcription [10–13]. Recent emerging evidence also suggests that circRNAs plays important roles in PCa, with the findings indicating their involvement in tumor cell proliferation, invasion, migration, and epithelial-mesenchymal transition (EMT) [14, 15]. However, the functions and mechanisms of most circRNAs remain largely unexplored in PCa metastasis.

The EMT process has been found to play a crucial role in PCa metastasis, wherein epithelial cells acquire mesenchymal phenotype along with loss of intercellular adhesion and conversion to migratory and invasive cells [16]. It is well-known that the induction and maintenance of EMT are closely controlled by signaling pathways and transcription factors, which involve a complex regulatory network of genes and noncoding RNAs [17]. Recent studies have also implicated circRNAs in cancer development, wherein they affect tumor cell migration, invasion, and cancer metastasis by regulating EMT [14, 18]. Previous studies have shown that the transcription factor ETS1 is involved in tumor progression and metastasis through EMT induction in various cancers, including hepatocellular carcinoma and colon cancer [19, 20].

In the present study, we identified circ_0004296 by RNA sequencing and qPCR and evaluated its role in PCa. The expression of circ_0004296 was decreased in PCa tissues, blood, and urine, and it was negatively associated with metastasis. Furthermore, gain and loss of function experiments in vitro and in vivo showed that circ_0004296 inhibited the proliferation, migration, invasion, and EMT of PCa cells. Mechanistically, circ_0004296 was localized in the nucleus, interacted with the RNA-binding protein (RBP) EIF4A3 to prevent nuclear export of host gene ETS1 mRNA, and subsequently inhibited its expression. Our study implied that circ_0004296 may serve as a specific biomarker and therapeutic target for patients with metastatic PCa (mPCa).

## Materials and methods

### Patient samples

All patients provided written informed consent. The present study was approved by the Ethics Committee of Shanghai Tenth People’s Hospital (approval number: SHSY-IEC-2014RES-36). Clinical pathological data were also collected through the hospital medical record system. Five pairs of PCa tissues and matched local metastatic lymph node tissues were obtained from radical resection of PCa and dissection of enlarged lymph nodes. Forty pairs of PCa tissues and matched adjacent normal tissues were obtained from radical resection of PCa. Forty-six urine specimens and 39 blood samples were obtained from patients with benign prostatic hyperplasia, localized PCa, and metastatic PCa. Tissue microarrays included 359 specimens from patients with PCa. These cases had corresponding follow-up information for an average of 32 months. All cases were confirmed by clinical and pathological diagnosis.

### Whole-transcriptome sequencing (RNA-seq)

Five pairs of PCa tissues and matched local metastatic lymph node tissues were used for RNA-seq. Total RNA was isolated using the QubitRNA Assay Kit (cat. Q32852; Life Technologies, USA) following the manufacturer’s protocol. RNA-seq and data analysis were performed by Oebiotech (Shanghai OEbiotech Co., Ltd., Shanghai, China).

### Cell culture

PCa cell lines (PC3, DU145, LNCaP, and 22RV-1) and the normal human prostate epithelial cell line RWPE-1 were purchased from the cell library of Shanghai Chinese Academy of Sciences (Shanghai, China). PCa cell lines were cultured in RPMI-1640 medium, supplemented with 10% fetal bovine serum (FBS), penicillin (100 U/ml), and streptomycin (100 μg/ml). RWPE-1 cells were cultured in Defined Keratinocyte SFM (1X) medium (Gibco, USA).

### Quantitative real-time PCR (qRT-PCR)

Total RNA for PCa tissues and cells were isolated using TRIzol as described previously [21]. Total RNA for urine and blood samples were isolated by magnetic bead nucleic acid extraction kit following the manufacturer’s instructions (Newskybio, China). According to the manufacturer’s instructions, RNA was reverse transcribed into cDNA using the HiScript® III 1st Strand cDNA Synthesis Kit (Vazyme, China), the qPCR was completed using ChamQ SYBR qPCR Master Mix Kit (Vazyme, China). The primers (Supplementary Table S1) were synthesized by Sangon (Shanghai, China).

### Cell transfection

PC3 and DU145 cells were transfected using Lipofectamine® 3000 (Invitrogen; Thermo Fisher Scientific, Inc., USA) following the manufacturer’s instructions. Plasmid and lentivirus expression vector constructs for overexpressing circ_0004296 were designed and synthesized by Zuorun (Shanghai, China) (Supplementary Table S1). Plasmid constructs for overexpressing ETS1 were designed and synthesized by Qihe (Shanghai, China). Small interfering RNAs (siRNAs) for the indicated gene sequences (Supplementary Table S1) were designed and synthesized by Ibsbio (Shanghai, China). DU145 cells were subsequently infected and selected with puromycin. The luciferase was then incorporated into DU145- circ_0004296 stable cells.

### Cell proliferation assays

Cell proliferation was assessed by the Cell Counting Kit-8 (CCK8) assay and expressed as colony formation as described previously [21]. Three independent replicates were set in each experiment; and all experiments were conducted in triplicate.

### Wound healing assay

Cell migration was measured by the wound healing assay as described previously [22]. Three independent replicates were set in each experiment; and all experiments were conducted in triplicate.

### Transwell assay

Cell migration and invasion were assessed by the Transwell migration and invasion assay as described previously [21]. Three independent replicates were set in each experiment; and all experiments were conducted in triplicate.

### Western blotting assay

Total protein was lysed from cells using radioimmunoprecipitation assay buffer (RIPA buffer; Beyotime, Shanghai, China). Protein quantification and western blotting assays were performed as described previously [21]. The following primary antibodies were used: anti-E-cadherin (WL01482, Wanleibio, China), anti-ETS1 (#14069, Cell Signaling Technology (CST), USA), anti-N-cadherin (WL01047, Wanleibio, China), anti-Snail (#3879, CST), anti-EIF4A3 (ab18057, Abcam, USA), and anti-Vimentin (ab8069, Abcam).

### Immunofluorescence staining

Protein expression was evaluated by immunofluorescence staining (IF) of PCa tissues and cells. Briefly, 5-μm paraffin-embedded cross-sections of tissues or cells uniformly grown on slides were fixed with 4% paraformaldehyde, permeabilized with 0.1% Triton-100, and blocked with 5% bovine serum albumin (BSA). Subsequently, tissues or cells were incubated with anti-E-cadherin (WL01482, Wanleibio), anti-EIF4A3 (ab18057, Abcam, USA), and anti-Vimentin (ab8069, Abcam) overnight at 4 °C. Then, the cells were treated with the corresponding secondary antibody after washing for three times. DAPI (Beyotime) staining was used for nuclear localization. Images were captured with a confocal microscope (Leica Microsystems, Mannheim, Germany).

### Animal study

All animal experiments were approved by the Animal Care and Use Committee of Shanghai Tenth People’s Hospital of Tongji University (approval number: SHDSYY-2014-3028). BABL/c male nude mice were housed under specific pathogen-free conditions. To develop a subcutaneous xenograft mouse model, twelve 4-week-old male nude mice were randomly divided into two groups, which were subcutaneously injected with 5 × 10^6^ DU145-vector or DU145-circ_0004296 cells. Tumor size was measured using a vernier caliper twice a week. Tumor volume was calculated as follows: 0.5 × length × width^2^. Five weeks later, mice were sacrificed, and all dissected tumor xenografts were weighed and subjected to immunohistochemistry (IHC). IHC was performed as described previously [22]. To develop an orthotopic xenograft mouse model, 8-week-old male nude mice received intraprostatic injections of 2 × 10^5^ DU145-vector or DU145-circ_0004296 cells in 10 μL of PBS, with six mice in each group. To develop a pulmonary metastatic model, 1 × 10^6^ DU145-vector or DU145-circ_0004296 cells were injected into the tail vein of 8-week-old male nude mice, with six mice in each group. The intraprostatic and metastatic tumors were observed weekly using the in vivo imaging system (IVIS). Mice were sacrificed when the weight loss was ≥10%, and the lung metastatic foci were examined microscopically by H&E staining.

### Fractionation of nuclear and cytoplasmic RNA

Nuclear and cytoplasmic RNA were extracted using the PARIS™ kit (Invitrogen, USA) following the manufacturer’s protocol. The qRT-PCR was used to detect RNA abundance in different cell fractions. The efficiency of nuclear and cytoplasmic RNA isolation was controlled by qRT-PCR using U6 and actin, respectively.

### Fractionation of nuclear and cytoplasmic protein

Nuclear and cytoplasmic protein fractionation assays were performed with nuclear and cytoplasmic protein extraction kit (Beyotime) following the manufacturer’s protocol. The purity of the subcellular fractions were detected using anti-GAPDH (ab18602, Abcam) and anti-Histone 3 (13108-1-AP, Proteintech, China) antibodies by western blotting assay.

### RNA pull-down assay

RNA pull-down assay for circ_0004296 and circ_0004296-binding proteins was performed using RNA pull-down kit (Bersinbio, Guangzhou, China). The biotinylated circ_0004296 probe used to pull down for circ_0004296 and RBPs was synthesized by GenePharma (Shanghai, China). DU145 cells were harvested and lysed with lysis buffer. The probes were incubated with streptavidin-coated magnetic beads to generate probe-coupled magnetic beads. The cell extract (800 μL) was incubated with magnetic beads coupled with the circ_0004296 probe or a negative control (NC) probe. Proteins pulled down by the probe were collected using a protein elution buffer. Mass spectrometry was used to identify differentially interacted proteins. Mass spectrometry and data analysis were performed by Wayenbio (Shanghai, China). The differential abundance of circ_0004296-binding proteins was confirmed by western blotting assay.

### RNA fluorescence in situ hybridization analysis (RNA-FISH)

RNA-FISH experiments were completed using a Ribo™ FISH kit (Ribobio, China) following the manufacturer’s instructions. The FISH probe of circ_0004296 was synthesized by GenePharma (Supplementary Table S1). An 18S RNA probe was used as a positive control (Ribobio).

### RNA immunoprecipitation assay (RIP)

RIP was completed with an EZ-Magna RIP kit (Millipore, Billerica, MA, USA). DU145 cells were harvested and lysed with lysis buffer. The cell extracts were incubated with magnetic beads coated with anti-EIF4A3 antibodies (ab180573, Abcam) or control (IgG, Beyotime). Subsequently, total RNA was extracted from the magnetic beads. qRT-PCR was used to determine the presence of ETS mRNA and circ_0004296.

### Bioinformatics analysis

The circRNA-binding proteins were predicted using the online program CircInteractome [23], Starbase [24], and CircFunBase [25].

### Statistical analysis

Data were analyzed and plotted using SPSS 25.0 (IBM, SPSS, Chicago, IL, USA) and GraphPad Prism 7 (GraphPad Prism Software Inc., San Diego, CA), which were shown as mean ± standard deviation (SD). Student’s *t*-test was used to evaluate the difference between two groups, while ANOVA test was used for more than two groups. All experiments were conducted in triplicate. Linear correlations were analyzed by Pearson’s correlation coefficient. The disease-free survival and biochemical recurrence-free survival was analyzed by Kaplan-Meier method and the log-rank test. *P* < 0.05 was considered to be statistically significant.

## Results

### Circ_0004296 is negatively associated with PCa metastasis

**Fig. 1:**
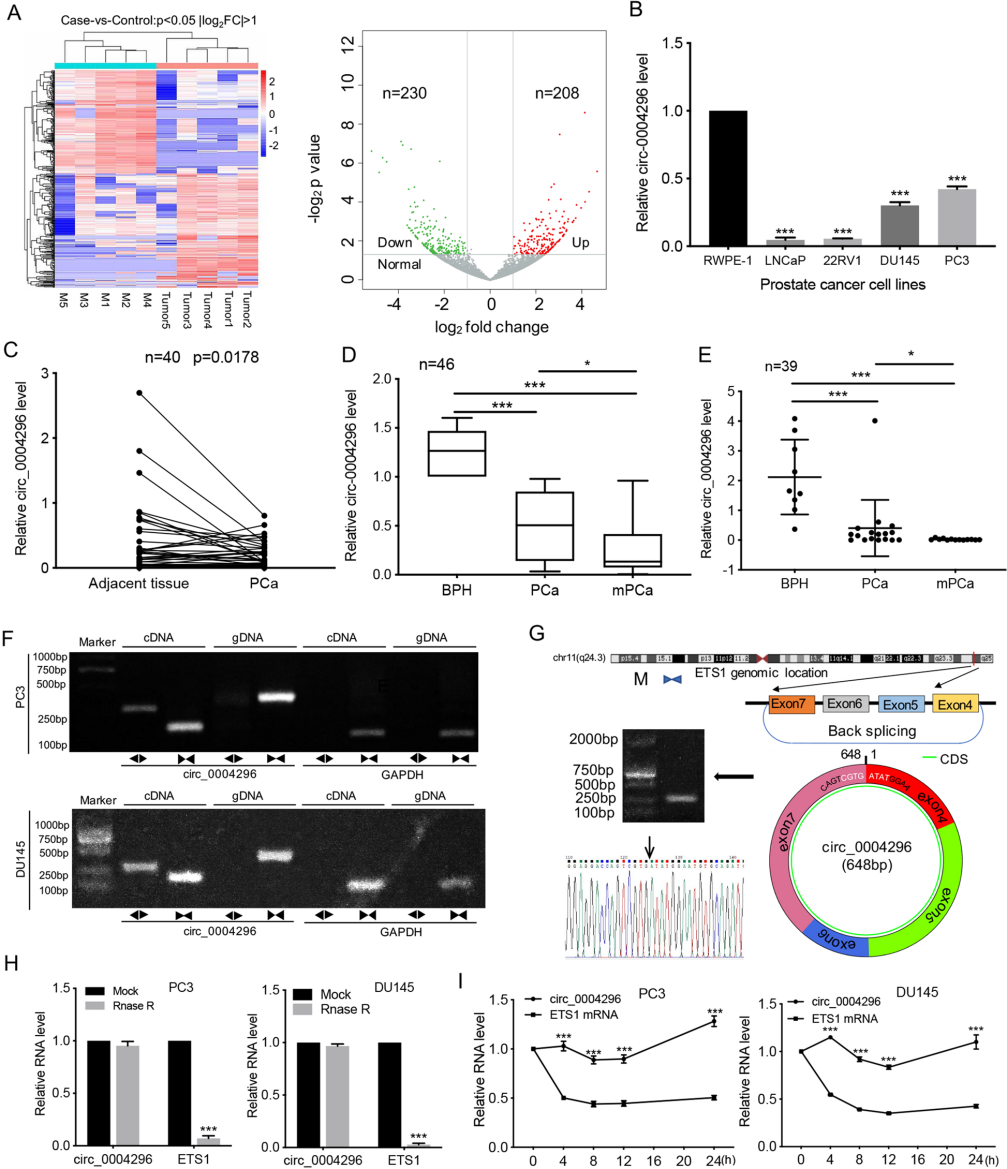
Hsa_circ_0004296 expression was significantly downregulated in PCa and negatively correlated with cancer metastasis. **A** Heatmap showing differentially expressed circRNAs (208 upregulated and 230 downregulated circRNAs) between five pairs of PCa and metastatic lymph node tissues (Cas-vs-Control: *p* value< 0.05 &|log2FC| > 1). **B** qRT-PCR showed that the expression of circ_0004296 in PCa cell lines was significantly downregulated. **C** qRT-PCR was used to detect the expression level of circ_0004296 in 40 paired PCa tissues and adjacent normal tissues. **D** qRT-PCR was used to detect the expression of circ_0004296 in urine from 46 patients with benign prostatic hyperplasia, localized PCa, and metastatic PCa. **E** qRT-PCR was used to detect the expression of circ_0004296 in plasma from 39 patients with nontumor, localized PCa, and metastatic PCa. **F** Northern blotting showed that circ_0004296 could be detected only in cDNA and not in gDNA. **G** Schematic map of circ_000429 formation and Sanger sequencing of the circ_0004296 splice junction. **H**, **I** RNase-R and actinomycin D treatment assays showed no change in the expression level of circ_0004296, while the expression of ETS1 was significantly decreased. *P < 0.05, **P < 0.01, ***P < 0.001

To investigate whether circRNAs are involved in PCa metastasis, five pairs of PCa tissues and matched local metastatic lymph node tissues were used for RNA-seq. The results showed a total of 22,676 circRNAs. Among these circRNAs, 208 upregulated circRNAs and 230 downregulated circRNAs were identified to show significant differential expression (fold change > 2.0 and *P* < 0.05) (Fig. 1A). Next, we designed divergent primers to detect six screened circRNA candidates in PCa cell lines, prostate normal epithelial cells, PCa tissues, and adjacent normal tissues (Supplementary Table S1). We chose circRNA_04265 (circBase ID: hsa_circ_0004296) as the candidate circRNA for further research, which is derived from back-splicing of exons 4, 5, 6, and 7 of host gene ETS1, with a length of 648 bp. The reasons were as followings: First, the circRNAs that are smaller than 2000 bp and annotated by the circBase database is selected. Then, circ_0004296 had relatively high expression abundance (Figure S1A); compared to that in prostate normal cells, circ_0004296 expression was significantly downregulated in all PCa cell lines (Fig. 1B and S1B-D); its host gene ETS1 was abnormally high expression and is an oncogenic gene in PCa, which suggests that the low expression of circ_0004296 may be functional. Consistently, compared to that in the adjacent normal tissue (*n* = 40), circ_0004296 was reduced in PCa tissues (Fig. 1C). Circ_0004296 was detected stably in urine (Fig. 1D) and blood plasma (Fig. 1E) from patients with benign prostatic hyperplasia, localized PCa, and metastatic PCa. Furthermore, the expression of circ_0004296 was negatively associated with distant metastasis (Fig. 1D and E). According to the consistent expression observed in PCa cell lines and PCa tissues by qRT-PCR, downregulated circ_0004296 might be involved in the occurrence and metastasis of PCa. We then designed divergent and convergent primers to amplify circ_0004296 and linear ETS1 mRNA by using cDNA and genomic DNA (gDNA) from PCa cells. Northern blotting showed that circ_0004296 can only be detected in the cDNA group, but not in the gDNA group (Fig. 1F). Sanger sequencing confirmed the back-spliced junction of circ_0004296 (Fig. 1G). To further confirm the stably expressed circ_0004296, PCa cells were treated with Rnase R and actinomycin D. The results of qRT-PCR showed that circ_0004296 had better stability than linear mRNA (Fig. 1H and I). These results suggested that circ_0004296 may play a suppressive role in PCa metastasis and serves as a noninvasive liquid biopsy biomarker.

### Circ_0004296 inhibited PCa cell proliferation, migration, invasion, and EMT in vitro

**Fig. 2:**
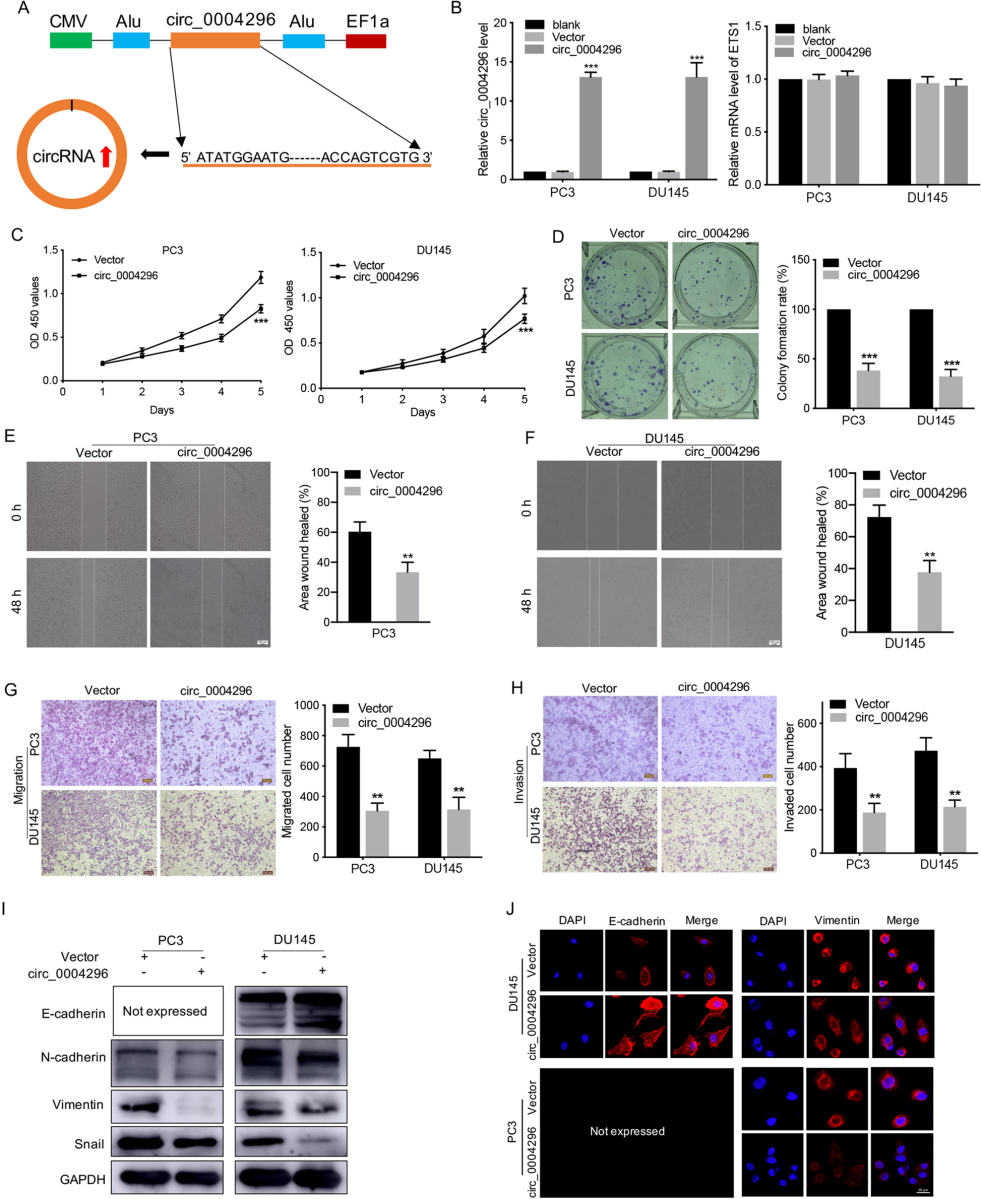
Overexpression of circ_0004296 inhibited the proliferation, migration, invasion, and EMT of PCa cells. **A** Schematic illustration of the circ_0004296 plasmid (pLenO-GTP- circ_0004296) or empty vector. **B** The expression of circ_0004296 and ETS1 was detected by qRT-PCR in PC3 and DU145 cells transfected with the circ_0004296 plasmid or empty vector. **C**, **D** CCK8 and colony formation assays were used to measure viability of PCa cells after circ_0004296 overexpression. **E**-**H** The migration and invasion abilities of PCa cells were analyzed by wound healing and Transwell assays for migration and invasion after circ_0004296 overexpression. Scale bars =100um. **I** Western blotting assay was performed to detect the expression of EMT-related proteins, namely E-cadherin, N-cadherin, Vimentin, and Snail, after circ_0004296 overexpression. **J** IF assays showed that circ_0004296 overexpression increased epithelial marker (E-cadherin) expression but reduced mesenchymal marker (Vimentin) expression in PCa cells. Scale bars =20um. *P < 0.05, **P < 0.01, ***P < 0.001

To investigate the function of circ_0004296 in PCa cells, the recombinant pLenO-GTP-circ_0004296-overexpression plasmid was constructed. The integrity of the linear circ_0004296 RNA sequence carried on the plasmid was confirmed by northern blotting and Sanger sequencing (Fig. 2A and S1E, Supplementary file 1). Overexpression of circ_0004296 significantly increased the level of circ_0004296 but had no effect on the linear mRNA level in PC3 and DU145 cells (Fig. 2B). The proliferation assay, scratch wound healing, and Transwell assay revealed that overexpression of circ_0004296 markedly inhibited proliferation, migration, and invasion of PC3 and DU145 cells compared to the negative control (Fig. 2C-H).

It is known that EMT plays a critical role in mediating the metastasis of human tumors, including PCa [16]. Next, IF and western blotting assays were performed to examine the impact of circ_0004296 on the expression of epithelial and mesenchymal markers. The results showed that circ_0004296 overexpression remarkably increased epithelial marker (E-cadherin) expression but reduced mesenchymal marker (N-cadherin, Vimentin and Snail) expression in PCa cells (Fig. 2I and J).

We also designed three siRNAs targeting the back-spliced junction (si-circ_0004296). The qRT-PCR results showed that siRNA#1 had the highest knockdown efficiency for circ_0004296 and had no effect on the linear mRNA level (Figure S2A and B). Furthermore, we found that circ_0004296 knockdown significantly promoted the proliferation, migration, and invasion of PCa cells (Figure S2C-H). Moreover, circ_0004296 knockdown remarkably decreased epithelial marker (E-cadherin) expression but increased mesenchymal marker (N-cadherin, Vimentin and Snail) expression in PCa cells (Figure S2I). Taken together, these findings demonstrated that circ_0004296 inhibited PCa cell proliferation, migration, invasion, and EMT.

### Circ_0004296 regulated host gene ETS1 expression through interaction with the RBP EIF4A3

**Fig. 3:**
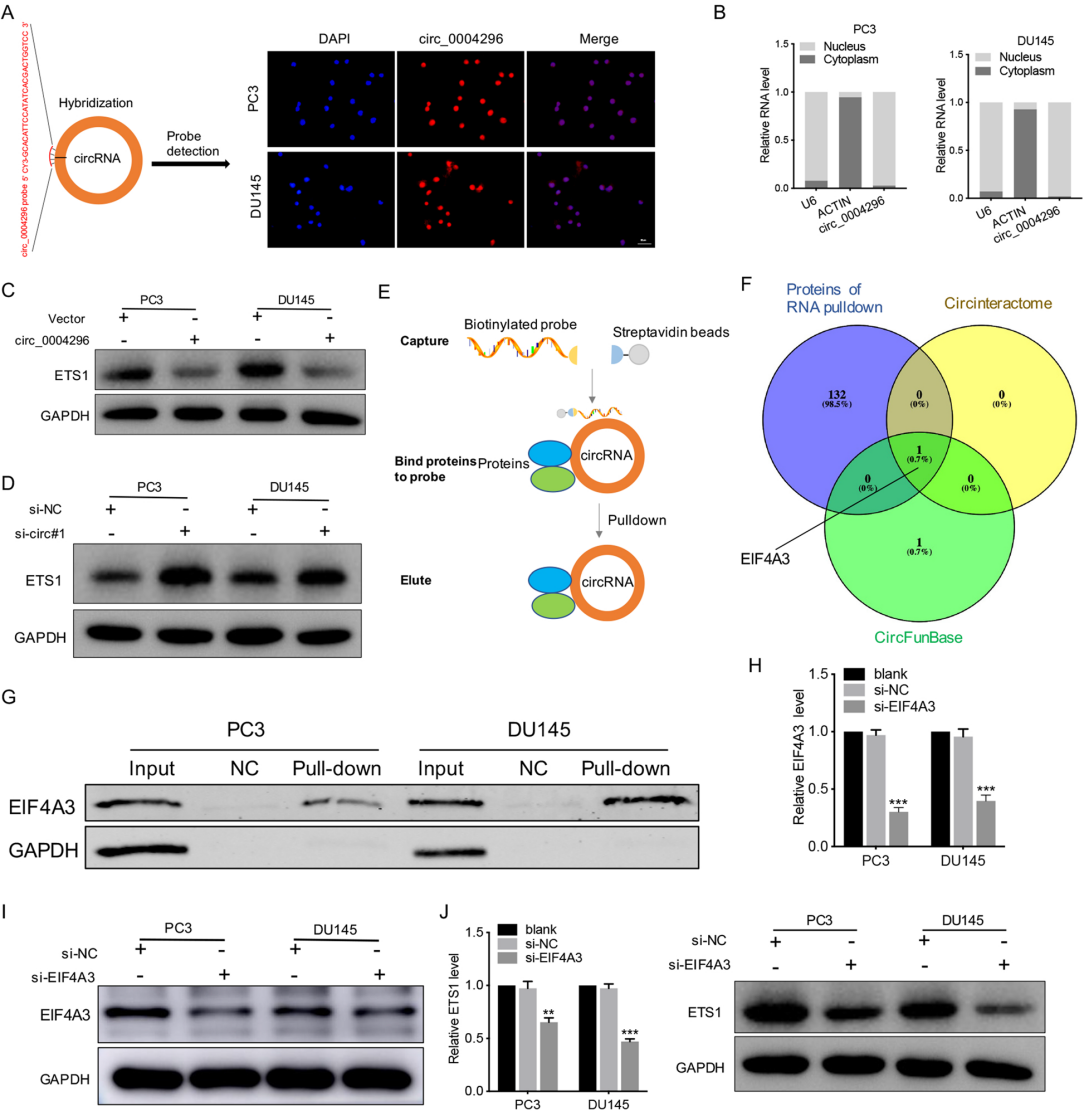
EIF4A3 protein mediated circ_0004296-induced inhibition of host gene ETS1 expression in PCa cells. **A**, **B** Intracellular distribution of circ_0004296 was detected by RNA-FISH and nuclear-plasma extraction assay. Scale bars =50um. **C** The circ_0004296 overexpression decreased the expression of the ETS1 protein. **D** circ_0004296 knockdown increased the expression of the ETS1 protein. **E** Schematic illustration of RNA pull-down for circ_0004296. **F** Schematic exhibiting the overlap for specific proteins bound by circ_0004296 via protein profiling and the potential target genes of circ_0004296 predicted by CircInteractome and CircFunBase. **G** RNA pull-down and western blotting assays confirmed the interaction of circ_0004296 with EIF4A3. **H** The efficiency of EIF4A3 knockdown at mRNA level. **I** The efficiency of EIF4A3 knockdown at protein level. **J** EIF4A3 knockdown decreased ETS1 expression. *P < 0.05, **P < 0.01, ***P < 0.001

**Fig. 4:**
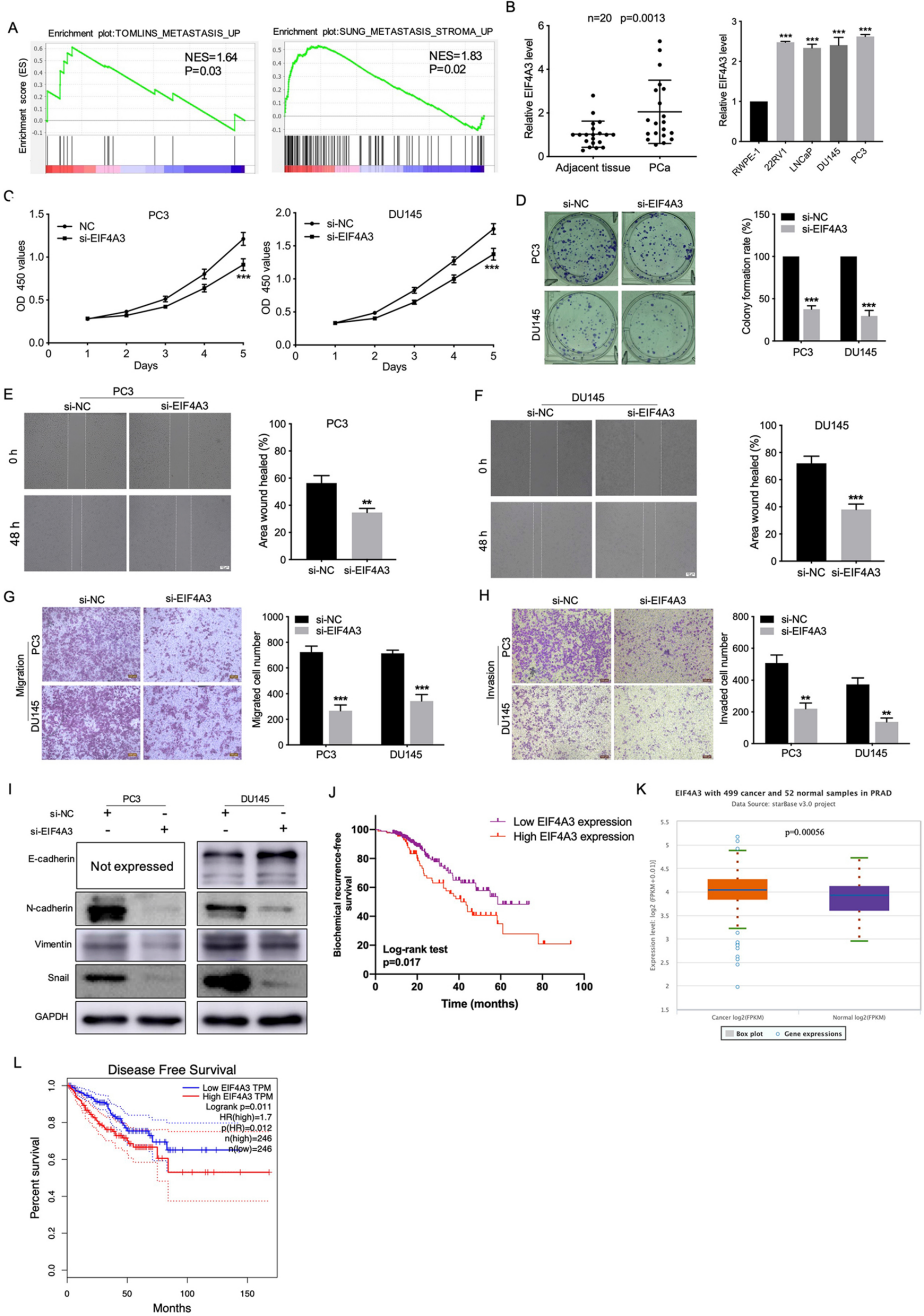
EIF4A3 knockdown promoted the proliferation, migration, invasion, and EMT induction of PCa cells. **A** GSEA of TOMLINS_METASTASIS_UP and SUNG_METASTASIS_STROMA_UP GSEA gene sets referred to as metastasis-related gene signatures; NES, normalized enrichment score. **B** EIF4A3 expression was detected by qRT-PCR in PCa cell lines, PCa tissues, and adjacent normal tissues. **C**, **D** CCK8 and colony formation assays were used to measure viability of PCa cells after EIF4A3 knockdown. **E**-**H** The migration and invasion abilities of PCa cells were analyzed by wound healing and Transwell assays for migration and invasion after EIF4A3 knockdown. Scale bars =100um. **I** Western blotting assay was performed to detect the expression of EMT-related proteins, namely E-cadherin, N-cadherin, Vimentin, and Snail, after EIF4A3 knockdown. **J** Kaplan-Meier analysis for different EIF4A3 protein expression in PCa patients. **K** EIF4A3 expression in PCa tissues and normal samples from the TCGA database. **L** EIF4A3 expression was associated with disease-free survival of 492 patients with PCa from the TCGA database. *P < 0.05, **P < 0.01, ***P < 0.001

**Fig. 5:**
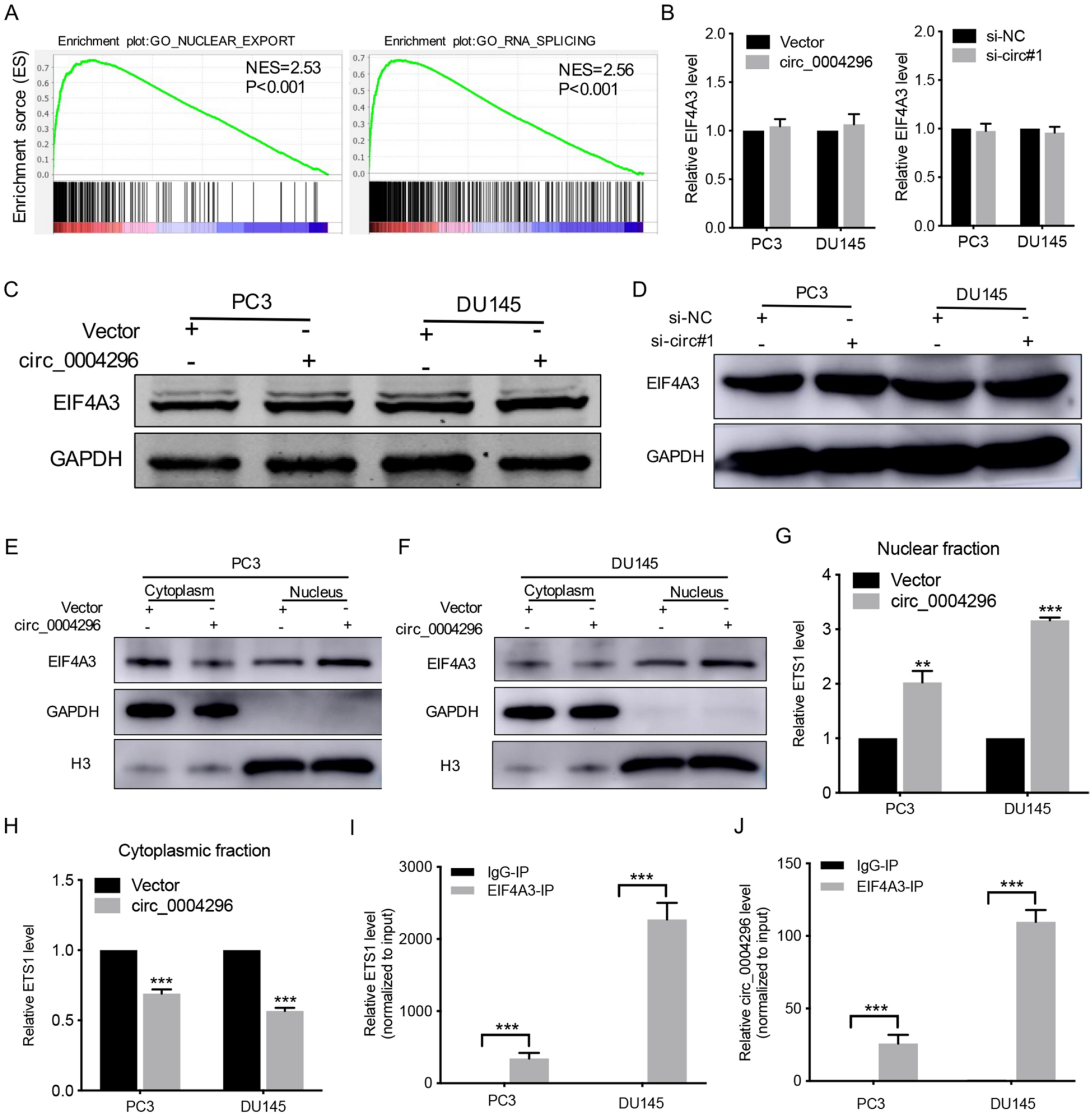
Circ_0004296 inhibited nuclear export of ETS1 mRNA through interaction with EIF4A3. **A** GSEA of GO_NUCLEAR_EXPORT and GO_RNA_SPLICING functional signatures in TCGA databases; NES, normalized enrichment score. **B**-**D** Knockdown or overexpression of circ_0004296 had no effect on the expression of EIF4A3 in PCa cells. **E**, **F** The levels of nuclear and cytoplasmic EIF4A3 protein were determined by western blotting assay after circ_0004296 overexpression. **G**, **H** The levels of nuclear and cytoplasmic ETS1 mRNA were determined by qRT-PCR after circ_0004296 overexpression. **I**, **J** RIP assay showed that EIF4A3 interacted with circ_0004296 and ETS1 mRNA in PCa cells. *P < 0.05, **P < 0.01, ***P < 0.001

First, the intracellular localization of circ_0004296 was detected in PCa cells by the RNA-FISH assay and the nuclear and cytoplasmic fractionation assay. The results showed the predominant nuclear distribution of circ_0004296 (Fig. 3A and B). Overexpression of circ_0004296 in PCa cell lines remarkably decreased the ETS1 protein level, but had no significant impact on the expression of ETS1 mRNA (Fig. 3C). Additionally, silencing circ_0004296 remarkably increased the ETS1 protein level, but had no obvious effect on the level of ETS1 mRNA (Fig. 3D). These results suggested that circ_0004296 regulated host gene ETS1 expression at the post-transcriptional level. Recent studies have shown the significance of circRNA-protein interaction [26]. Some RBPs have been identified to have an interplay with circRNAs through the regulation of gene expression in cancers [10, 15]. Given that circ_0004296 is mainly distributed in the nucleus, we investigated whether circ_0004296 regulated host gene ETS1 expression through interaction with an RBP by performing the RNA pull-down assay and mass spectrometry analysis (Fig. 3E, F and G). The specific proteins that interacted with circ_0004296 are presented in Supplementary Table S2. From the prediction results of CircInteractome, Starbase, and CircFunBase, we selected EIF4A3 as the downstream binding protein of circ_0004296 (Fig. 3F). RNA pull-down and western blotting assays confirmed the interaction of circ_0004296 with EIF4A3 (Fig. 3G). Silencing EIF4A3 remarkably decreased ETS1 protein and mRNA levels (Fig. 3H-J). These results suggested that circ_0004296 regulated host gene ETS1 expression by interacting with EIF4A3. To determine the potential functions of EIF4A3 in PCa, GSEA was performed to link the published molecular signature databases for PCa metastasis to high versus low EIF4A3 expression in TCGA databases (Gene Set: TOMLINS_METASTASIS_UP and SUNG_METASTASIS_STROMA_UP). GSEA showed that cell metastasis was significantly enriched in the PCa group, which strongly suggested that EIF4A3 was closely related to PCa metastasis (Fig. 4A). We also found that EIF4A3 was upregulated in PCa tissues and cells (Fig. 4B). Silencing EIF4A3 suppressed PCa cell proliferation, migration, invasion, and EMT (Fig. 4C-I). Kaplan-Meier analysis showed that EIF4A3 upregulation was associated with shorter biochemical recurrence-free survival of 359 patients with PCa (log-rank test, *p* = 0.017, Fig. 4J). Moreover, EIF4A3 expression was significantly upregulated in PCa tissues compared to that in normal samples from the TCGA database (Fig. 4K). Subsequently, Kaplan-Meier analysis suggested that EIF4A3 upregulation was associated with shorter disease-free survival of 492 patients with PCa from the TCGA database (log-rank test, *p* = 0.011, Fig. 4L). These results supported that EIF4A3 protein mediated circ_0004296 inhibition of PCa cell growth and metastasis. To investigate the function of ETS1 in PCa cells, three siRNAs targeting ETS1 were designed and the recombinant ETS1-overexpression plasmid was constructed. The results of western bolt showed that siRNA#1 had the highest knockdown efficiency for ETS1 (Figure S3B) and ETS1 expression was upregulated (Figure S3C). The proliferation assay and Transwell assay revealed that silencing ETS1 suppressed PCa cell proliferation and migration (Figure S3D, E). The proliferation assay and Transwell assay revealed that overexpression of ETS1 promoted proliferation and migration of PC3 and DU145 cells compared to the negative control (Figure S3F, G). Kaplan-Meier analysis showed that ETS1 upregulation was associated with shorter biochemical recurrence-free survival of 343 patients with PCa (log-rank test, *p* < 0.001, Figure S3H). The rescue experiment was performed to assess the effect of the circ_0004296/EIF4A3/ETS1 axis on PCa cells. The results showed that overexpression of ETS1 in pLenO-GTP-circ_0004296-overexpression cells could restore the function of circ_0004296-mediating inhibition of proliferation and migration (Fig. 5A and B).

Taken together, these findings demonstrated that circ_0004296 inhibited PCa cell proliferation, migration, invasion, and EMT through the EIF4A3/ETS1 axis.

### Circ_0004296 inhibited nuclear export of ETS1 mRNA through binding to EIF4A3

**Fig. 6:**
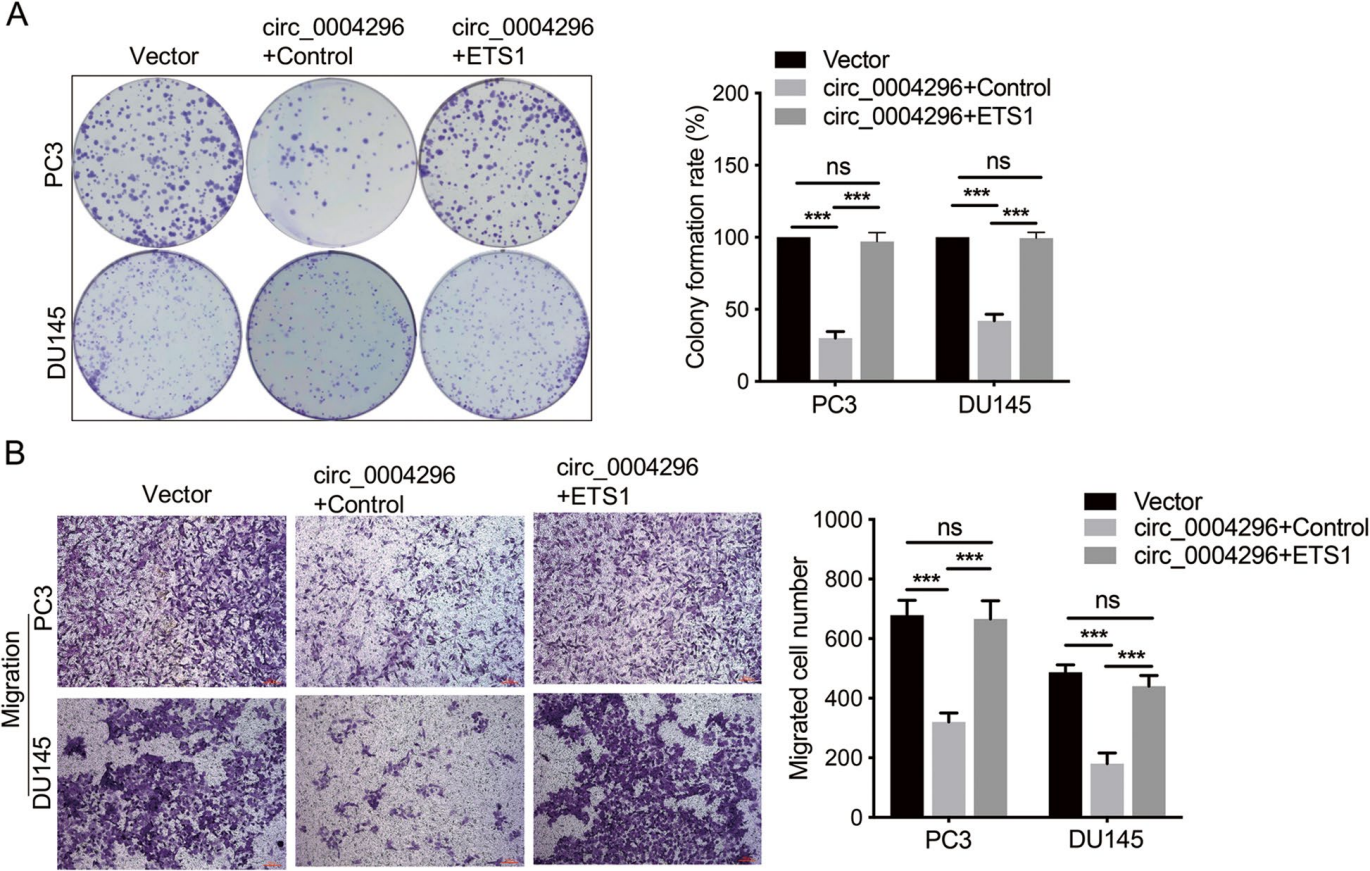
circ_0004296 inhibited PCa cell proliferation and migration through the EIF4A3/ETS1 axis. **A** Colony formation assays were used to measure viability of circ_0004296-overexpressing stable PCa cells transfected with ETS1 plasmid. **B** The migration abilities of PCa cells were analyzed by Transwell assays for migration circ_0004296-overexpressing stable PCa cells transfected with ETS1 plasmid. Scale bars =100um. *P < 0.05, **P < 0.01, ***P < 0.001

Furthermore, GSEA supported that the mRNA splicing and mRNA export functional signatures were significantly enriched for the high versus low EIF4A3 expression in TCGA databases (Fig. 6A); this finding strongly suggested that EIF4A3 was closely related to ETS1 expression at the post-transcriptional level. To investigate the potential regulatory relationship between circ_0004296 and EIF4A3, the expression of EIF4A3 was measured after silencing or overexpression of circ_0004296. The results showed that silencing or overexpression of circ_0004296 had no effect on the expression of EIF4A3 in PC3 and DU145 cells (Fig. 6B-D). Additionally, the knockdown of EIF4A3 had no significant impact on circ_0004296 expression in PCa cells (Figure S3A). Previous studies have shown that the RBP EIF4A3, which is mainly located in the nucleus and is the core component of the exon junction complex (EJC), can facilitate nucleocytoplasmic transport of mRNA [27, 28]. Thus, we speculated that circ_0004296 might be involved in the regulation of nuclear ETS1 mRNA export in PCa cells. We therefore subjected circ_0004296-overexpressing cells to nuclear and cytoplasmic fractionation. The nuclear and cytoplasmic levels of EIF4A3 protein were detected by western blotting assay. The nuclear and cytoplasmic levels of ETS1 mRNA were determined by qRT-PCR. In circ_0004296-upregulated cells, the proportions of nuclear EIF4A3 protein and nuclear ETS1 mRNA were increased (Fig. 6E-H). RIP assay further demonstrated that EIF4A3 interacted with circ_0004296 and ETS1 mRNA in DU145 cells (Fig. 6I and J); this finding is consistent with the prediction of EIF4A3 binding to ETS1 mRNA by Starbase from HITS-CLIP seq [24]. These data suggested that circ_0004296 efficiently inhibited ETS1 mRNA nuclear export by promoting EIF4A3 retention in the nucleus.

### Circ_0004296 inhibited PCa growth and metastasis in vivo

**Fig. 7:**
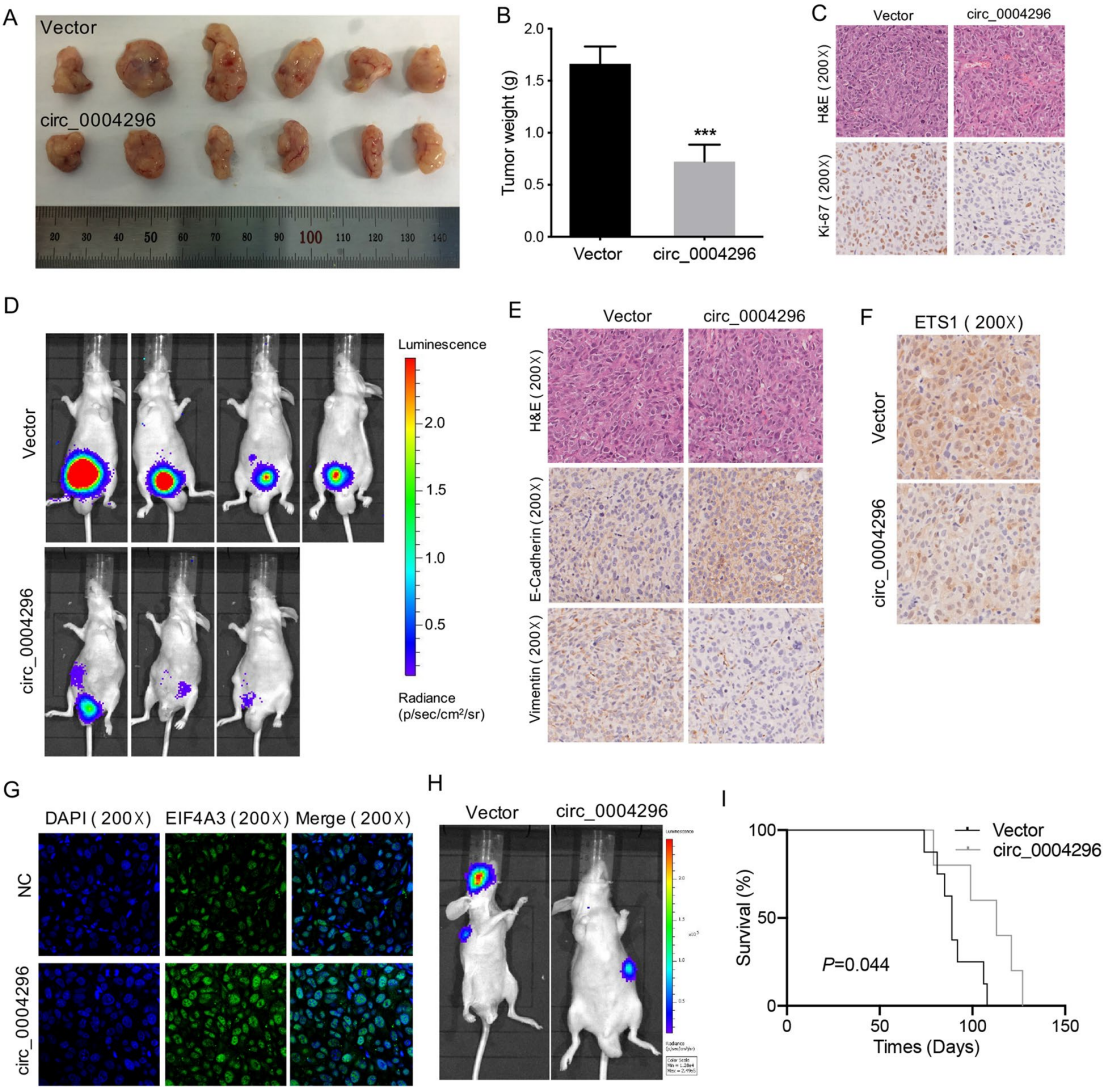
Circ_0004296 inhibited PCa growth and metastasis in vivo. **A**, **B** The subcutaneous xenograft mouse model showed that overexpression of circ_0004296 inhibited PCa growth in vivo (DU145-Vector, *n* = 6; DU145-circ_0004296, *n* = 6). **C** H&E staining and IHC assay showed that circ_0004296 overexpression downregulated the Ki67 protein level (magnification 200×). **D** The orthotopic xenograft mouse model showed that overexpression of circ_0004296 suppressed PCa growth and pelvic peritoneal invasion. **E** IHC assay showed that circ_0004296 overexpression remarkably downregulated mesenchymal marker (Vimentin) protein level but upregulated epithelial marker (E-cadherin) protein level (magnification 200×). **F** IHC assay showed that overexpression of circ_0004296 remarkably reduced the ETS1 protein level (magnification 200×). **G** IF assay showed that the expression of EIF4A3 protein from the circ_0004296 overexpression group was increased in the nucleus as compared to that in the control group. (magnification 200×). **H** The in vivo tail vein injection model showed that overexpression of circ_0004296 suppressed metastasis of PCa. **I** Kaplan–Meier analysis showing survival of mice with intravenous xenograft (DU145-Vector, *n* = 8; DU145-circ_0004296, *n* = 5). *P* value was determined using a log-rank test. *P < 0.05, **P < 0.01, ***P < 0.001

To further examine the effect of circ_0004296 in vivo, we constructed luciferase-labeled stably DU145-overexpressing circ_0004296 and luciferase-labeled DU145-vector cells (Figure S3I and J). The results of the subcutaneous xenograft mouse model showed that overexpression of circ_0004296 inhibited PCa growth in vivo (Fig. 6A and B). The xenograft tumor was subjected to IHC assay. The results showed that the circ_0004296 overexpression downregulated the Ki67 protein level (Fig. 7C). The results of the orthotopic xenograft mouse model showed that overexpression of circ_0004296 suppressed PCa growth and pelvic peritoneal invasion (Fig. 7D). The results of IHC assay showed that circ_0004296 overexpression remarkably downregulated the mesenchymal marker (Vimentin) protein level but upregulated the epithelial marker (E-cadherin) protein level (Fig. 7E). Additionally, IHC showed that overexpression of circ_0004296 remarkably reduced the ETS1 protein level (Fig. 7F). IF was performed to confirm the change in the subcellular localization of EIF4A3 protein in xenograft tumor, and the results showed that the expression of EIF4A3 protein from the circ_0004296 overexpression group was increased in the nucleus as compared to that in the control group (Fig. 7G). Furthermore, The results of the in vivo tail vein injection model showed that overexpression of circ_0004296 suppressed metastases of PCa (Fig. 7H). The longevity of the mice post-injection further indicated circ_0004296 inhibited PCa metastasis in the intravenous xenograft assay (Fig. 7I). Taken together, these findings in vivo also demonstrated that circ_0004296 inhibited PCa cell growth and metastasis through the EIF4A3/ETS1 axis.

## Discussion

**Fig. 8:**
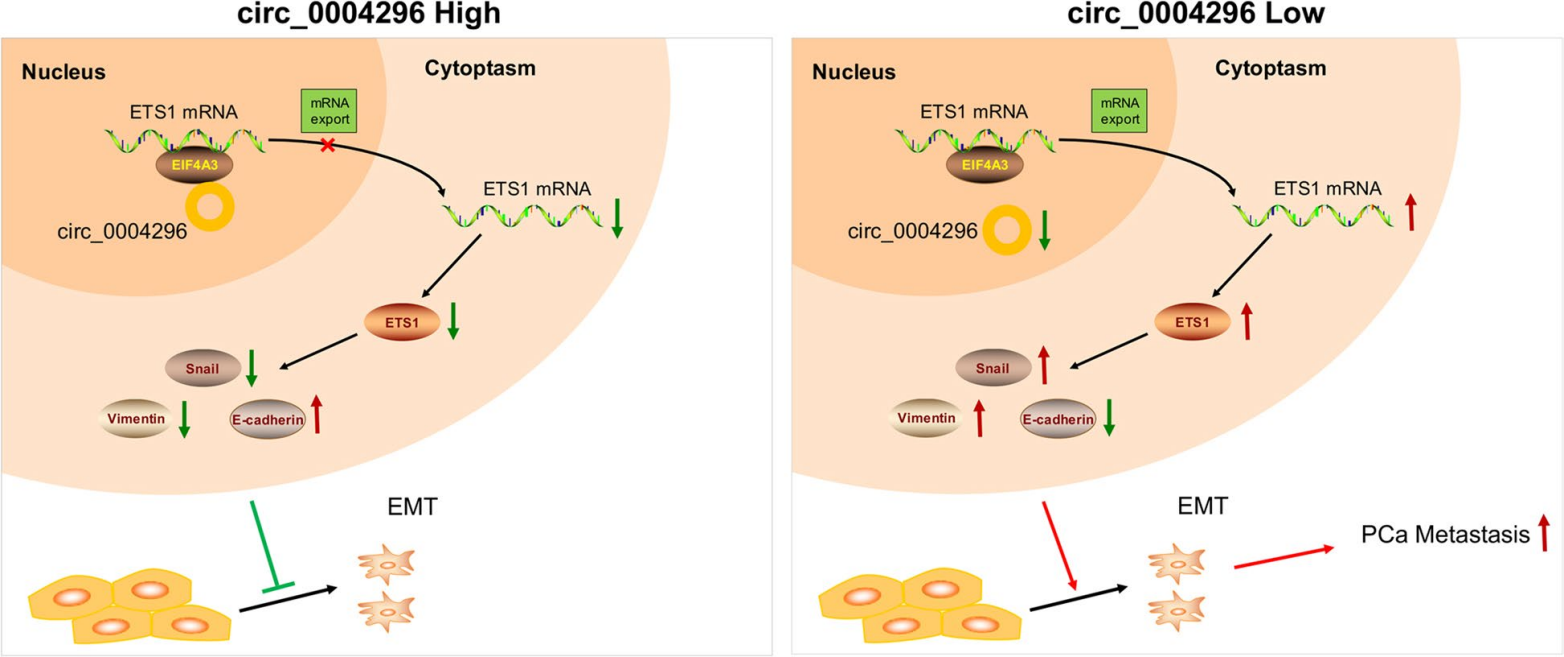
A schematic model for the potential roles of the circ_0004296/EIF4A3/ETS1 axis in PCa metastasis. Circ_0004296 efficiently decreases nuclear export of ETS1 mRNA by increasing EIF4A3 retention in the nucleus and subsequently inhibits EMT and metastasis of PCa cells

The present study first demonstrated that circ_0004296 was downregulated in PCa tissues and cell lines. We also confirmed that circ_0004296 was stably downregulated in the blood plasma and urine of patients with metastatic PCa, which predicted distant metastasis. Further in vitro and in vivo experiments confirmed that circ_0004296 inhibited the proliferation, migration, invasion, and EMT of PCa cells. Mechanistically, we showed that circ_0004296 was mainly located in the nucleus, and it interacted with RBP EIF4A3 and regulated the host gene ETS1 expression to inhibit the function of PCa cells. Furthermore, circ_0004296 efficiently inhibited nuclear export of ETS1 mRNA by promoting EIF4A3 retention in the nucleus (Fig. 8). Taken together, our study suggested that circ_0004296 might serve as a noninvasive liquid biopsy biomarker and a novel therapeutic target in human PCa.

Increasing evidence suggests that circRNAs are aberrantly expressed in multiple human tumors, and they play vital roles in tumor growth, tumor progression, metastasis, and drug resistance [6]. Previous studies have shown that circ-AMOTL1L is expressed at a low level in PCa tissues and inhibit invasion and migration of PCa cells [14]. In contrast, circ0005276 and circ-AGO2 are highly expressed in PCa and play a role in promoting cancer cell proliferation and migration [15, 29]. However, the biological functions of most circRNAs in PCa are currently unknown. In the present study, we screened 443 differentially expressed circRNAs in metastatic lymph node tissues and PCa tissues from the same patient. From the screening process, we selected a new circRNA, namely circ_0004296, whose host gene plays a key role in various tumors [30]. exoRBase, a web-accessible database (http://www.exoRBase.org), is a repository of circular RNA (circRNA), long non-coding RNA (lncRNA) and messenger RNA (mRNA) derived from RNA-seq data analyses of human blood exosomes, indicating that circulating circRNAs may be diagnostic and prognostic biomarkers [31]. CircSHKBP1 was abundant in serum exosomes and was similarly expressed in the serum and tumors of gastric cancer patients, suggested that circSHKBP1 is a stable biomarker for gastric cancer diagnosis and prognosis [32]. Circ-CCAC1 plays a vital role in cholangiocarcinoma tumorigenesis and metastasis, and exosomal circ-CCAC1 may be an important biomarker/therapeutic target for cholangiocarcinoma [33]. In this study, the downregulation of circ_0004296 in PCa cell lines and clinical samples suggested that circ_0004296 may play a suppressive role in PCa metastasis and serves as a noninvasive liquid biopsy biomarker.

It is known that EMT is important for the progression and metastasis of cancer cells. The primary PCa epithelial cells gain migratory and invasive properties by undergoing EMT [16]. Importantly, our further functional studies revealed that overexpression of circ_0004296 remarkably inhibited proliferation, migration, invasion, and EMT of PC3 and DU145 cells. In line with this result, circ_0004296 knockdown significantly enhanced proliferation, migration, invasion, and EMT of PCa cells. Consistent with these findings, the in vivo experiment demonstrated that overexpression of circ_0004296 markedly inhibited the growth, metastasis, and EMT of PCa cells. Thus, these results strongly supported the suppressive role of circ_0004296 in PCa metastasis.

Although the role of circRNAs in cancers has been widely reported, the downstream regulatory mechanisms remain largely obscure. To date, most circRNAs have been reported to play an important role by acting as ceRNAs to inhibit miRNAs [8, 9]. However, in the present study, circ_0004296 was found to be mainly located in the nucleus of PCa cells, indicating circ_0004296 might not be an miRNA sponge or translated into peptides. At present, only few studies have reported on the mechanism of circRNAs located in the nucleus. Interestingly, circRNAs produced by precursor mRNA back-splicing of their host genes have potential regulatory relationship with their host genes. Circ0005276 plays a crucial role in regulating host gene XIAP expression at the transcriptional level by interacting with the RBP FUS in PCa [15]. Recently, circPOK was found to bind the RNA/DNA-binding proteins ILF2 and ILF3 mainly in the nucleus and potentiate their regulatory activities in mediating both mRNA transcription and stability [34]. It was demonstrated that circSMARCA5 maily located in the nucleus can suppress the expression of host gene SMARCA5 by binding to its parent gene locus to form an R-loop, which results in transcriptional pausing [35]. In our present study, we found that circ_0004296 was mainly located in the nucleus and regulated the host gene ETS1 expression at the post-transcriptional level. Previous studies have shown that the transcription factor ETS1 is involved in tumor progression and metastasis through the transcriptional regulation of EMT-related genes in several cancers [19, 20], including PCa [36]. Recent studies have reported that circRNAs may play crucial roles in regulating gene expression at the transcriptional level by interacting with RBPs [15, 34, 37]. Next, we screened the proteins that specifically bind to circ_0004296 by performing RNA pull-down and mass spectrometry analyses, and bioinformatics analysis was performed to predict RBPs that may bind to circ_0004296. RNA pull-down and western blotting assays confirmed that circ_0004296 interacted with the RBP EIF4A3, a core component of the EJC complex [28]. EIF4A3 has been reported to promote the production of a majority of circRNAs [38–40]. On the contrary, a recent study found that EIF4A3 could inhibit the expression of circ_100290 in gastric cancer [41]. In the present study, knockdown of EIF4A3 had no significant impact on circ_0004296 expression in PCa cells. Silencing EIF4A3 remarkably decreased ETS1 mRNA and protein levels. Moreover, RIP assay showed that EIF4A3 interacted with circ_0004296 and ETS1 mRNA. The EJC complex is a critical chromatin remodeler and regulates gene expression [27, 28, 42]. It is known that EIF4A3 plays essential roles in mRNA splicing, nuclear mRNA export, subcellular mRNA localization, translation efficiency, and nonsense-mediated mRNA decay [42]. Previous research showed that circSEMA5A increased the expression of host gene SEMA5A by recruiting EIF4A3 to enhance mRNA stability of SEMA5A, and thereby promoted BC angiogenesis [43]. Additionally, hsa_circ_0030042 promoted the cytoplasmic localization of EIF4A3 and inhibits FOXO1 and beclin1 expression by obstructing the interaction between EIF4A3 and their mRNAs [44]. Emerging studies have demonstrated that through direct interaction, circRNAs can modulate the nuclear localization of proteins as well as their corresponding biological functions. Circ-Amotl1 can promote the nuclear localization of c-Myc, STAT3, PDK1, and AKT1 by direct interaction with them [45]. Through its different regions, circ-Dnmt1 can bind to p53 and Auf1, and facilitate their nuclear localization [46]. Circ-Foxo3 is predominantly localized in the cytoplasm and interacts with ID1, E2F1, HIF1α, and FAK, leading to their cytoplasm retention [11]. Through the interaction with HuR, protein, circAGO2 facilitates its translocation from the nucleus to the cytoplasm [29]. Furthermore, circ_0004296-overexpressing cells were subjected to nuclear and cytoplasmic fractionation. circ_0004296-overexpressing cells showed retention of EIF4A3 protein and ETS1 mRNA in the nucleus as compare to the control group. Thus, our study revealed a novel role of the circRNA circ_0004296 in PCa, wherein it directly interacted with EIF4A3 and suppressed nuclear export of ETS1 mRNA. Although EIF4A3 has been reported to be closely related to tumorigenesis, such as tumorigenesis of colorectal cancer [47] and pancreatic adenocarcinoma [48], its role in PCa has not yet been elucidated. Our present study found that EIF4A3 was highly expressed in PCa, and knockdown of EIF4A3 significantly suppressed proliferation, migration, invasion, and EMT of PCa cells. Thus, our results illustrated for the first time that EIF4A3 contributes to PCa development.

The upstream regulatory mechanisms of circRNA downregulation should thus be addressed in future research. The specific motif sites of EIF4A3 binding to circ_0004296 and ETS1 mRNA have not been elucidated and require further studies.

## Conclusion

Overall, the present study demonstrated that circ_0004296 was downregulated in PCa and inhibited cancer metastasis by suppressing EMT. We also confirmed circ_0004296 was stably downregulated in the blood plasma and urine of patients with PCa, thus indicating its potential for precision targeted treatment. Mechanistically, EIF4A3 protein mediated circ_0004296-induced inhibition of host gene ETS1 expression at the post-transcriptional level. Furthermore, our research revealed a novel role of the circRNA circ_0004296, wherein it directly interacted with EIF4A3, suppressed nuclear export of ETS1 mRNA, and subsequently suppressed EMT in PCa. Thus, the present study provides novel insights into the potential roles of the circ_0004296/EIF4A3/ETS1 axis in the therapeutic management of PCa.

## Supplementary Information

**Additional file 1: File S1.** The Sanger sequencing of the linear circ_0004296 RNA sequence carried on the plasmid.

**Additional file 2: Table S1.** Primers, siRNAs, probes, and plasmid used in the present study.

**Additional file 3: Table S2.** The specific RBPs interacted with circ_0004296.

**Additional file 4: Figure S1.** A. The expression abundance of six screened circRNA candidates. B. qRT-PCR showed the expression of circ_0001708 (circRNA_19382) in PCa cell lines. C. qRT-PCR showed the expression of circ_0002842 (circRNA_22335) in PCa cell lines. D. qRT-PCR showed the expression of circ_0004390 (circRNA_00938) in PCa cell lines. E. Northern blotting assay confirmed the integrity of the linear circ_0004296 RNA sequence carried on the plasmid.

**Additional file 5: Figure S2.** The circ_0004296 knockdown promoted the proliferation, migration, invasion, and EMT induction of PCa cells. A. Schematic illustration of designed siRNAs (siRNA#1, siRNA#2, and siRNA#3) targeting the back-splice junction of circ_0004296. B. The expression of circ_0004296 and ETS1 was detected by qRT-PCR in PC3 and DU145 cells transfected with siRNA-circ_0004296 or si-NC. C, D. CCK8 and colony formation assays were performed to measure the viability of PCa cells after circ_0004296 knockdown. E-H. The migration and invasion abilities of PCa cells were analyzed by wound healing and Transwell assays for migration and invasion after circ_0004296 knockdown. Scale bars =100um. I. Western blotting assay was performed to detected the expression of EMT-related proteins, namely E-cadherin, N-cadherin, Vimentin, and Snail, after circ_0004296 knockdown. J. Protein profiling was used to screen specific proteins bound by circ_0004296 in PCa cells. *P < 0.05, **P < 0.01, ***P < 0.001.

**Additional file 6: Figure S3.** A. Knockdown of EIF4A3 had no effect on circ_0004296 expression. B. The efficiency of ETS1 knockdown at protein level. C. The efficiency of ETS1 overexpression at protein level. D. Colony formation assays were used to measure viability of PCa cells after ETS1 knockdown. E. The migration abilities of PCa cells were analyzed by Transwell assays for migration after ETS1 knockdown. Scale bars =100um. F. Colony formation assays were used to measure viability of PCa cells after ETS1 overexpression. G. The migration abilities of PCa cells were analyzed by Transwell assays for migration after ETS1 overexpression. Scale bars =100um. H. Kaplan-Meier analysis for different ETS1 protein expression in PCa patients. I, J. Luciferase-labeled stably DU145-overexpressing circ_0004296 and luciferase-labeled DU145-vector cells were constructed. Scale bars =200um. *P < 0.05, **P < 0.01, ***P < 0.001.

**Additional file 7.**

## Data Availability

The mass spectrometry analysis data are included in the manuscript. All other data are available from the corresponding authors upon reasonable request.

## Abbreviations

circRNA: Circular RNA
PCa: Prostate cancer
EMT: Epithelial-mesenchymal transition
RBP: RNA-binding protein
mPCa: Metastatic PCa
qRT-PCR: Real-time quantitative PCR
CCK8: Cell Counting Kit-8
IVIS: In vivo imaging system
RNA-FISH: RNA fluorescence in situ hybridization
RIP: RNA immunoprecipitation
siRNA: Small interfering RNA
