# Supplementary material for: Hsa_circ_0004296 inhibits metastasis of prostate cancer by interacting with EIF4A3 to prevent nuclear export of ETS1 mRNA
Source: J Exp Clin Cancer Res. 2021 Oct 25;40:336. doi: 10.1186/s13046-021-02138-8 (PMC8543852; doi:10.1186/s13046-021-02138-8)

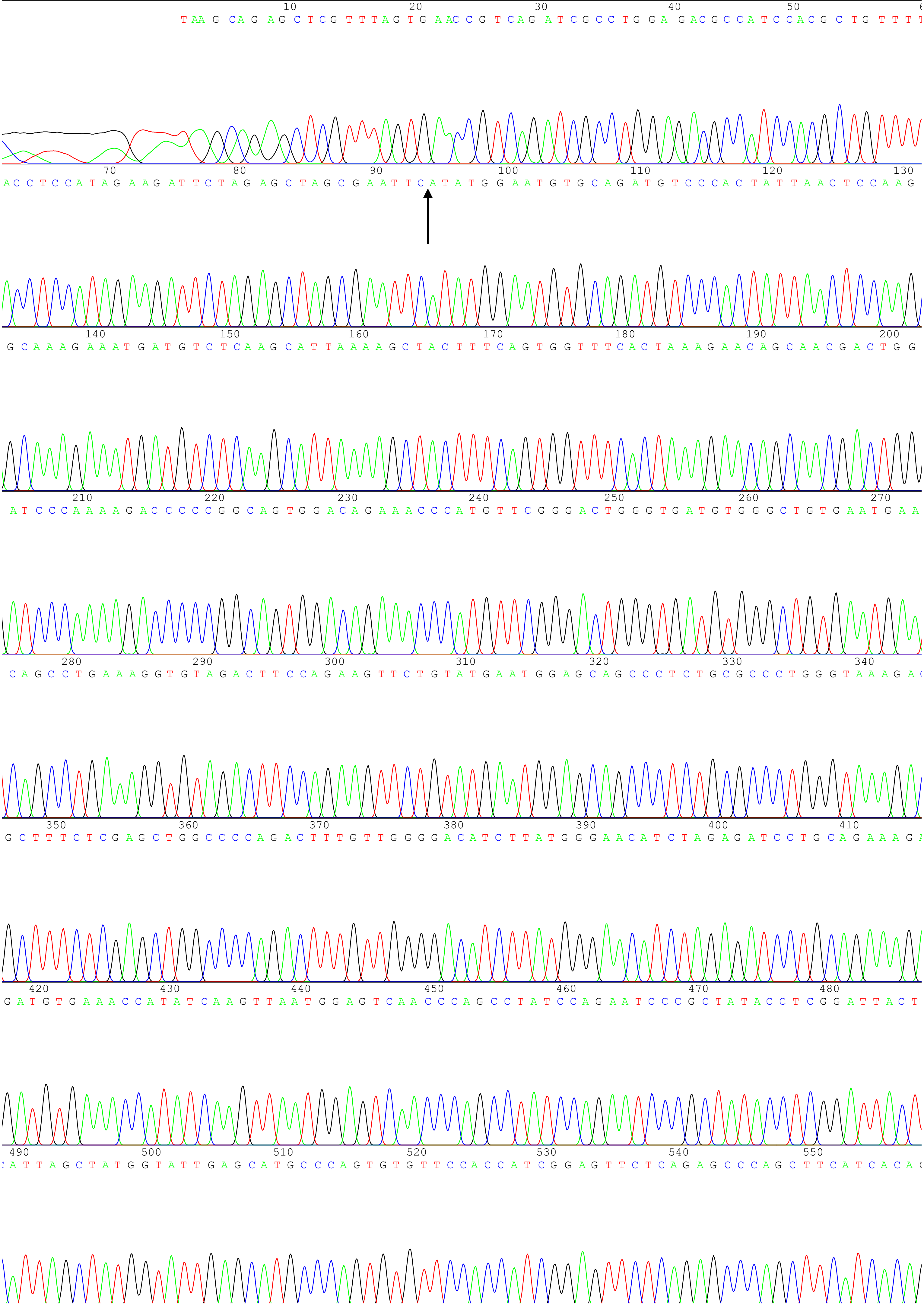
10 20 30 40 50 6
TAAGCAGAGCTCGTTTAGTGAACCGTCAGATCGCCTGGAGACGCCATCCACGCTGTTTT
70 80 90 100 110 120 130
ACCTCCATAGAAGATTCTAGAGCTAGCGAATTCATATGGAATGTGCAGATGTCCCACTATTAACTCCAAG
140 150 160 170 180 190 200
GCAAAGAAATGATGTCTCAAGCATTAAAAGCTACTTTCAGTGGTTTCACTAAAGAACAGCAACGACTGG
210 220 230 240 250 260 270
ATCCAAAAGACCCCGGCAGTGGACAGAAACCCATGTTCGGGACTGGGTGATGTGGGCTGTGAATGAA
280 290 300 310 320 330 340
CAGCCTGAAAGGTGTAGACTTCCAGAAGTTCTGTATGAATGGAGCAGCCCTCTGCGCCCTGGGTAAAGA
350 360 370 380 390 400 410
GCTTTCTCGAGCTGGCCCCAGACTTTGTTGGGGACATCTTATGGGAACATCTAGAGATCCTGCAGAAAGA
420 430 440 450 460 470 480
GATGTGAAACCATATCAAGTTAATGGAGTCAACCCAGCCTATCCAGAATCCCGCTATACCTCGGATTACT
490 500 510 520 530 540 550
ATTAGCTATGGTATTGAGCATGCCCAGTGTGTTCCACCATCGGAGTTCTCAGAGCCCAGCTTCATCACA

0 570 580 590 600 610 620 6
G T C C T A T C A G A C G C T C C A T C C C A T C A G C T C G G A A G A G C T C C T C T C C C T C A A G T A T G A G A A T G A C T A C C C C

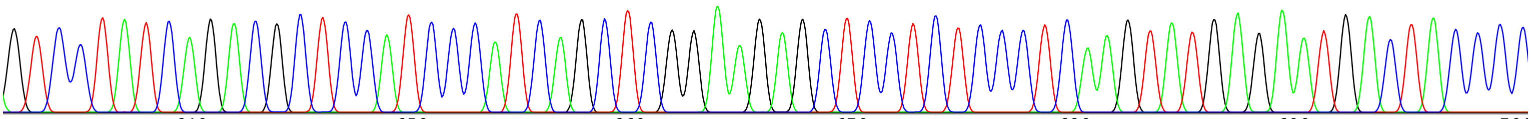

640 650 660 670 680 690 700
C G G T C A T T C T C C G A G A C C C T C T C C A G A C A G A C A C C T T G C A G A A T G A C T A C T T T G C T A T C A A A C A A G A A G T

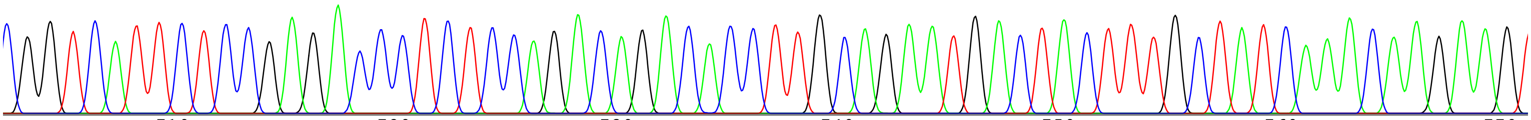

710 720 730 740 750 760 770
G T C A C C C C A G A C A A C A T G T G C A T G G G G A G G A C C A G T C G T G G G A T C C G C G G C C G C G A A G G A T C T G C G A T C

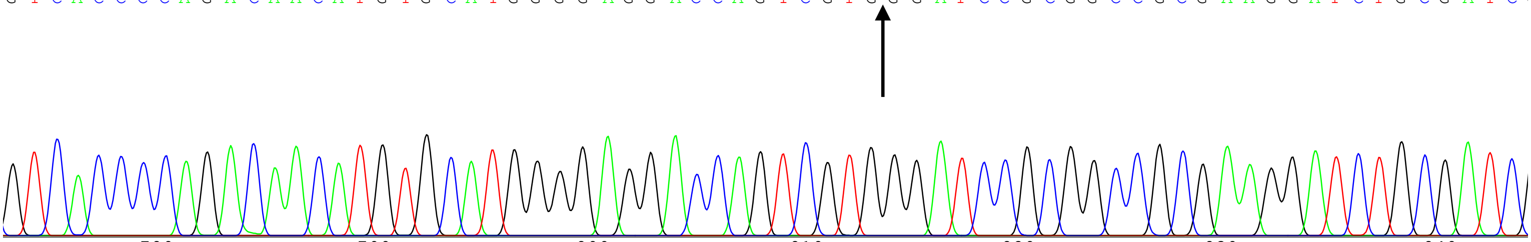

780 790 800 810 820 830 840
T C C G G T G C C C G T C A G T G G G C A G A G C G C A C A T C G C C C A C A G T C C C C G A G A A G T T G G G GG G G A G G G G T C G G C A

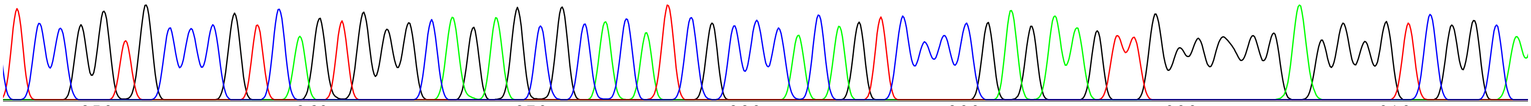

850 860 870 880 890 900 910
T G A A C G G G T G C C T A G A G A A G G T G G C G C G G G G T A A A C T G G G A A A G T G A T G T C G T G T A C T G G C T C C G C C T T T

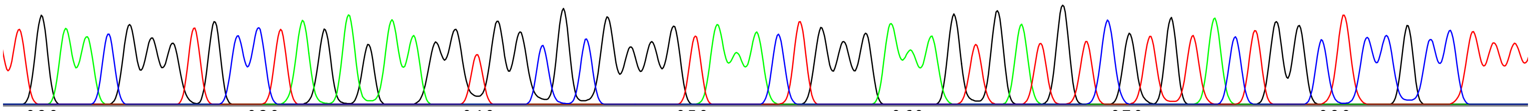

920 930 940 950 960 970 980
C C C G A G G G T G G G G G A G A A C C G T A T A T A A G T G C A G T A G T C G C C G T G A A C G T T C T T T T T C G C A A C G G G T T T G C

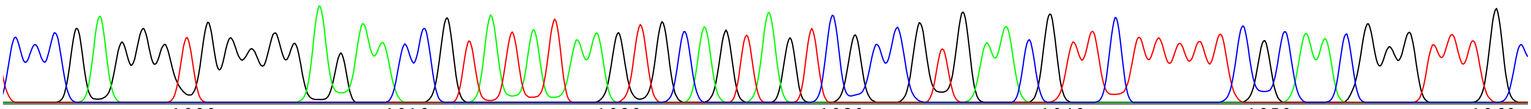

1000 1010 1020 1030 1040 1050 1060
C C A G A A C A C A G C T G A A G C T T C G A G G G G C T C G C A T C T C T C T T C A C G C G C C G C C G C C C T A C C T G A G G C G C C A T

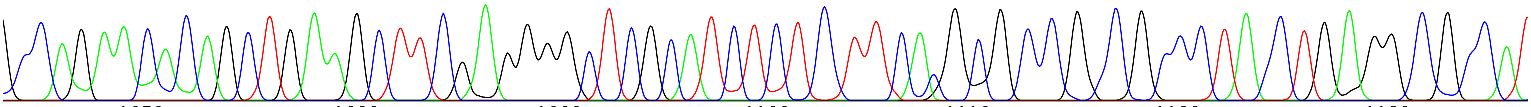

1070 1080 1090 1100 1110 1120 1130
C A C G C C G G T T G A G T C G C G T T C T G C C G C C T C C C G C C T G T G G T G C C T C C T G A A C T G C G T C C G C C G T C T A G G T A A G T

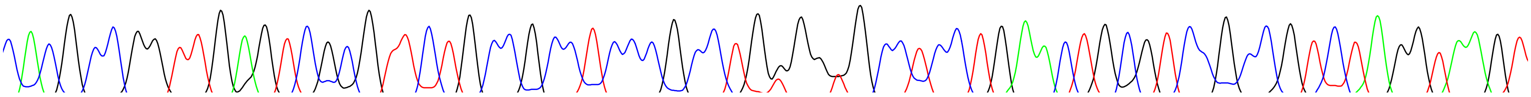

1140 1150 1160

' AA A G C T C A G G T C G A G A C G G G C C T T T G

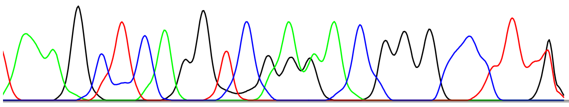

Supplement: Supplementary file 1 — Additional file 1: File S1. The Sanger sequencing of the linear circ_0004296 RNA sequence carried on the plasmid. [file 13046_2021_2138_MOESM1_ESM.pdf]
