# Supplementary figures and images for: Hsa_circ_0004296 inhibits metastasis of prostate cancer by interacting with EIF4A3 to prevent nuclear export of ETS1 mRNA
Source: J Exp Clin Cancer Res. 2021 Oct 25;40:336. doi: 10.1186/s13046-021-02138-8 (PMC8543852; doi:10.1186/s13046-021-02138-8)

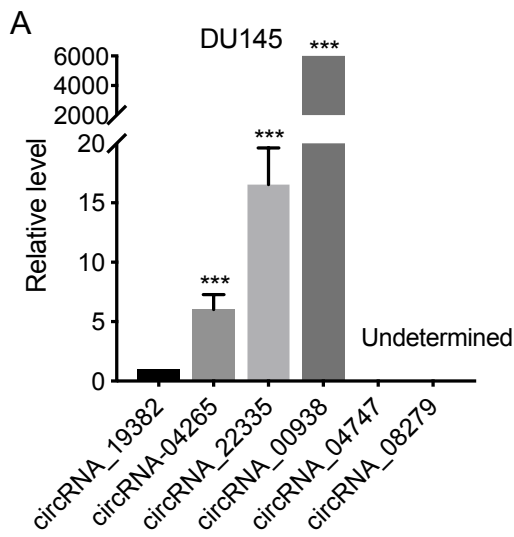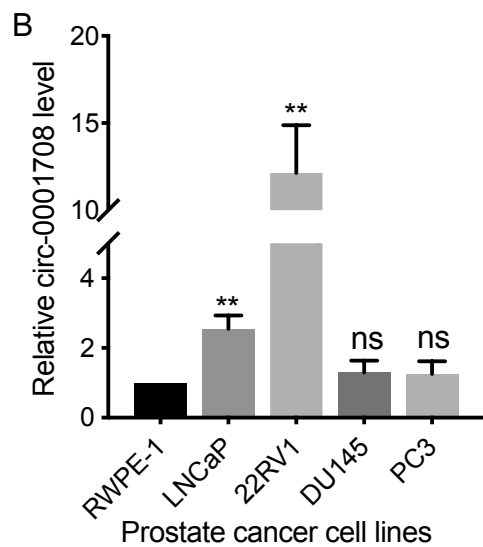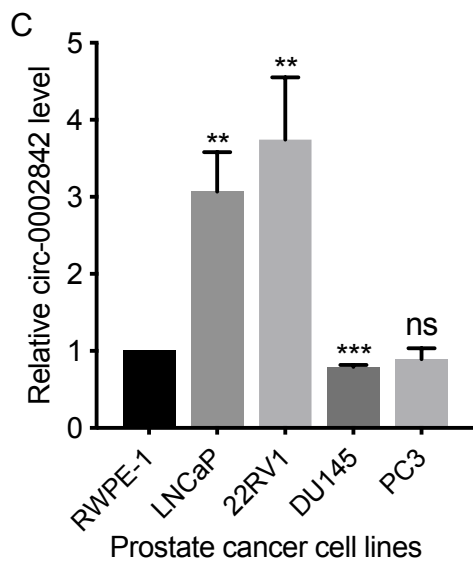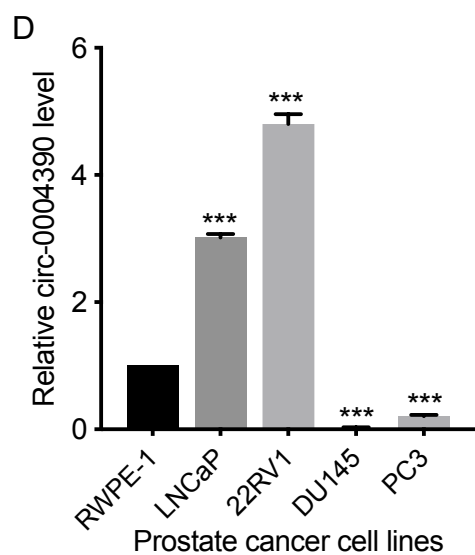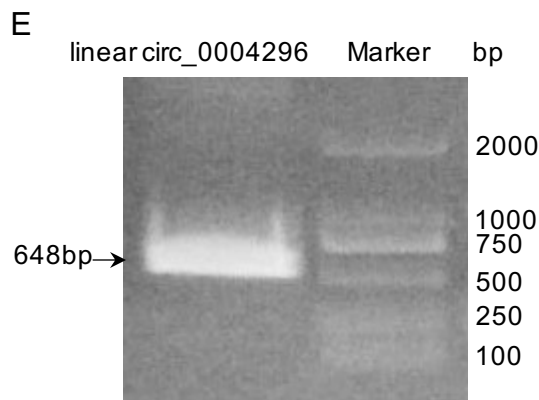

Supplement: Supplementary file 4 — Additional file 4: Figure S1. A. The expression abundance of six screened circRNA candidates. B. qRT-PCR showed the expression of circ_0001708 (circRNA_19382) in PCa cell lines. C. qRT-PCR showed the expression of circ_0002842 (circRNA_22335) in PCa cell lines. D. qRT-PCR showed the expression of circ_0004390 (circRNA_00938) in PCa cell lines. E. Northern blotting assay confirmed the integrity of the linear circ_0004296 RNA sequence carried on the plasmid. [file 13046_2021_2138_MOESM4_ESM.pdf]

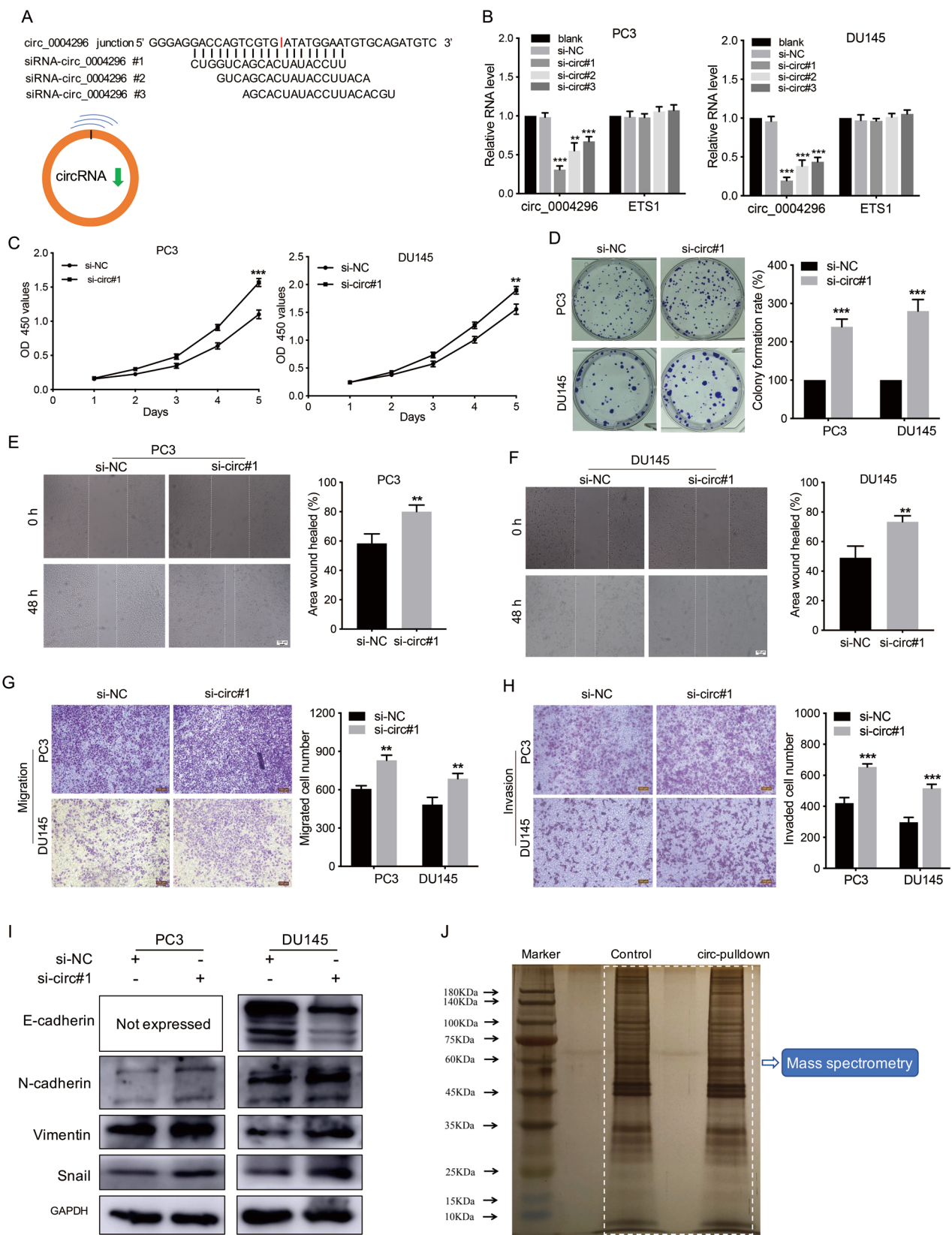

Supplement: Supplementary file 5 — Additional file 5: Figure S2. The circ_0004296 knockdown promoted the proliferation, migration, invasion, and EMT induction of PCa cells. A. Schematic illustration of designed siRNAs (siRNA#1, siRNA#2, and siRNA#3) targeting the back-splice junction of circ_0004296. B. The expression of circ_0004296 and ETS1 was detected by qRT-PCR in PC3 and DU145 cells transfected with siRNA-circ_0004296 or si-NC. C, D. CCK8 and colony formation assays were performed to measure the viability of PCa cells after circ_0004296 knockdown. E-H. The migration and invasion abilities of PCa cells were analyzed by wound healing and Transwell assays for migration and invasion after circ_0004296 knockdown. Scale bars =100um. I. Western blotting assay was performed to detected the expression of EMT-related proteins, namely E-cadherin, N-cadherin, Vimentin, and Snail, after circ_0004296 knockdown. J. Protein profiling was used to screen specific proteins bound by circ_0004296 in PCa cells. *P < 0.05, **P < 0.01, ***P < 0.001. [file 13046_2021_2138_MOESM5_ESM.pdf]

A

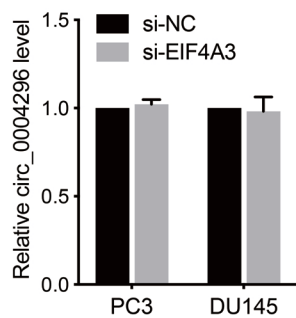

B

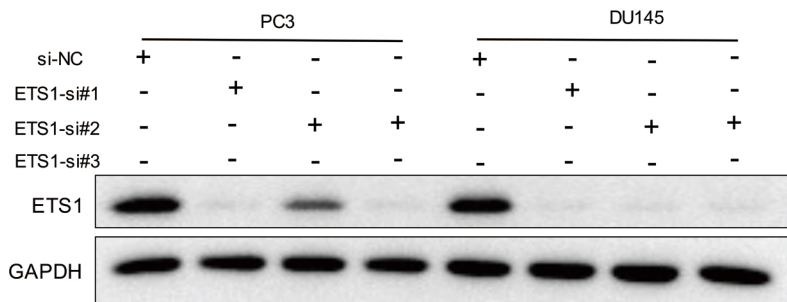

C

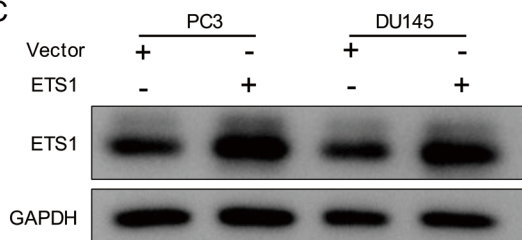

D

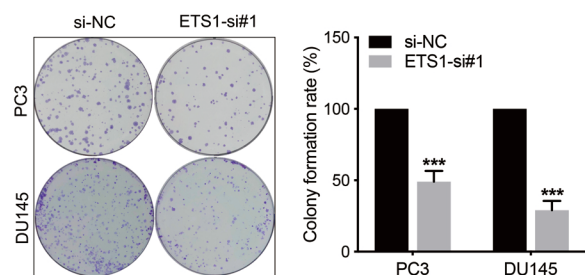

E

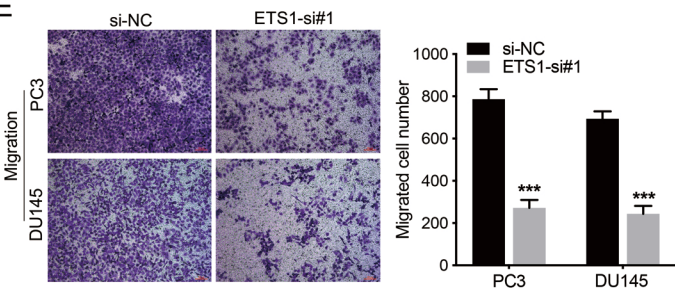

F

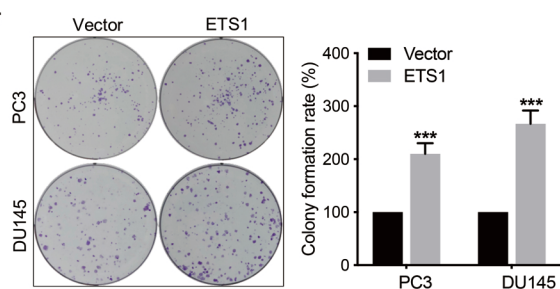

G

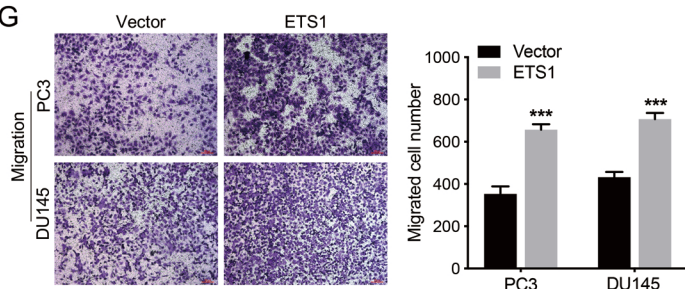

H

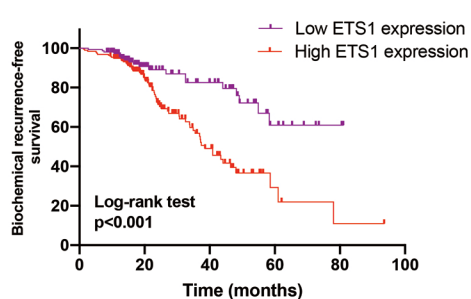

I

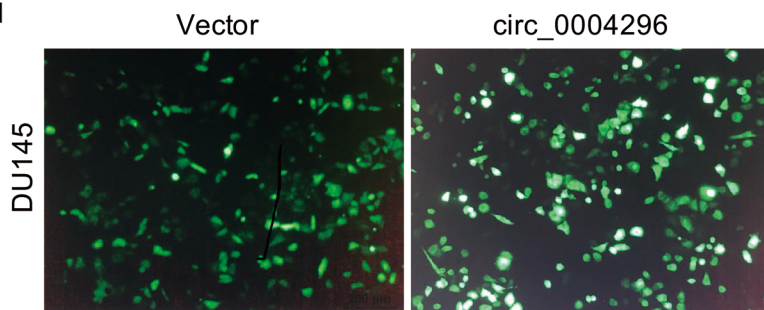

J

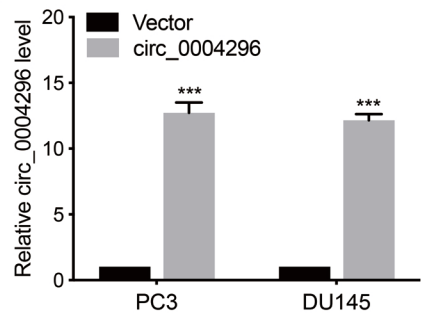

Supplement: Supplementary file 6 — Additional file 6: Figure S3. A. Knockdown of EIF4A3 had no effect on circ_0004296 expression. B. The efficiency of ETS1 knockdown at protein level. C. The efficiency of ETS1 overexpression at protein level. D. Colony formation assays were used to measure viability of PCa cells after ETS1 knockdown. E. The migration abilities of PCa cells were analyzed by Transwell assays for migration after ETS1 knockdown. Scale bars =100um. F. Colony formation assays were used to measure viability of PCa cells after ETS1 overexpression. G. The migration abilities of PCa cells were analyzed by Transwell assays for migration after ETS1 overexpression. Scale bars =100um. H. Kaplan-Meier analysis for different ETS1 protein expression in PCa patients. I, J. Luciferase-labeled stably DU145-overexpressing circ_0004296 and luciferase-labeled DU145-vector cells were constructed. Scale bars =200um. *P < 0.05, **P < 0.01, ***P < 0.001. [file 13046_2021_2138_MOESM6_ESM.pdf]
