# Supplementary material for: Hsa_circ_0004296 inhibits metastasis of prostate cancer by interacting with EIF4A3 to prevent nuclear export of ETS1 mRNA
Source: J Exp Clin Cancer Res. 2021 Oct 25;40:336. doi: 10.1186/s13046-021-02138-8 (PMC8543852; doi:10.1186/s13046-021-02138-8)
Supplement: Supplementary file 7 — Additional file 7. [file 13046_2021_2138_MOESM7_ESM.zip › 22RV1 STR.pdf]

22Rv1 细胞 STR 鉴定报告

一、 材料处理和检验方法

取适量 **22Rv1** 细胞(1×10^6)使用 PureLink® Genomic DNA Mini Kit（美国 Life K182001）提前基因组 DNA，采用 PowerPlex®18D 系统（美国 Promega DC1802）试剂盒进行扩增，在 ABI3500 Genetic Analyzer（美国 Life3500）进行检测。

二、 检测结果

实验中阴性及阳性对照结果均正确。

22Rv1 细胞株的 STR 位点和 Amelogenin 位点的基因分型结果见附表，分型图谱见附图。

三、 分析说明

22Rv1 细胞株基因组 DNA 扩增后图谱清晰，分型结果良好。

四、 检验结论

1. **22Rv1** 细胞株 DNA 进行细胞 STR 分型结果显示，细胞株中未发现人类细胞交叉污染。
2. 该细胞株 DNA 分型在 ATCC 细胞库中找到与其细胞分型 94%相匹配的细胞株，细胞株名称为 **22Rv1**。

附表 1：细胞株 22Rv1 的 STR 位点和 Amelogenin 位点的基因分型结果

细胞 22Rv1（图片编号为 PC143）			
Marker	***Allele 1***	***Allele 2***	***Allele 3***
D3S1358	15	15	
**THO1**	6	9.3	
D21S11	30	30	
D18S51	13	14	
Penta E	5	13	
**D5S818**	11	13	
**D13S317**	9	12	
**D7S820**	9	10	11
**D16S539**	12	12	
**CSF1PO**	10	11	
Penta D	9	12	
**AMEL**	X	Y	
**vWA**	15	21	
D8S1179	13	14	
**TPOX**	8	8	
FGA	19	20	23
D6S1043	16	16	
D2S1338	17	18	
D12S391	18	25	
D19S433	13	14	
D1S1656	17	18	19

附图 1：ATCC 官网 22Rv1 细胞 STR 位点信息

22Rv1 (ATCC® CRL-2505™)

Organism: **Homo sapiens, human** / Cell Type: **epithelial** / Tissue: **prostate** / Dis

GENERAL INFORMATION | CHARACTERISTICS | CULTURE METHOD | SPECIFICATIONS

STR Profile

Amelogenin: X,Y
CSF1PO: 10,11
D13S317: 9,12
D16S539: 12
D5S818: 11,12,13
D7S820: 9,10,11
THO1: 6,9.3
TPOX: 8
vWA: 15,21

网站：www.procell.com.cn
电话：400-650-3656
邮箱：sales@procell.com.cn

武汉普诺赛生命科技有限公司

Procell Life Science&Technology Co,.Ltd.

附图 2：22Rv1 细胞 STR 位点和 Amelogenin 位点的基因分型结果

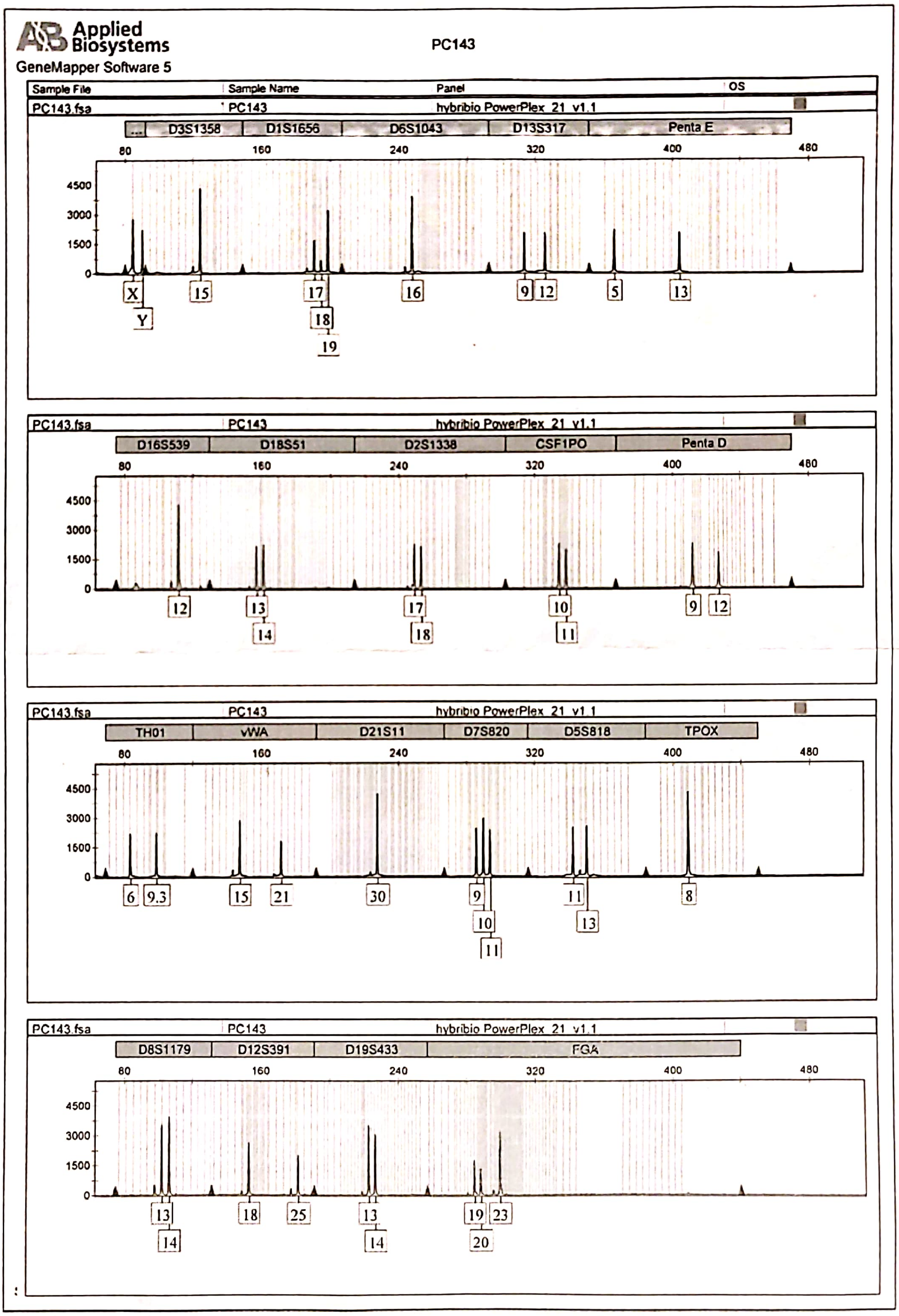

网站：www.procell.com.cn
电话：400-650-3656
邮箱：sales@procell.com.cn
