# Supplementary material for: Hsa_circ_0004296 inhibits metastasis of prostate cancer by interacting with EIF4A3 to prevent nuclear export of ETS1 mRNA
Source: J Exp Clin Cancer Res. 2021 Oct 25;40:336. doi: 10.1186/s13046-021-02138-8 (PMC8543852; doi:10.1186/s13046-021-02138-8)

细胞遗传质量鉴定检测

Cell Line Authentication Service

STR 基因型检测报告

送检单位：赛百慷
检品名称：细胞系
委托单位：上海翼和应用生物技术有限公司
报告日期：2019-09-16

上海翼和应用生物技术有限公司 联系地址：上海市漕河泾开发区桂平路 471 号桂果园 8 号楼 4F 室
网址：http://www.biowing.com.cn/邮箱：biowing@vip.163.com 服务热线：021-33559491

报 告 说 明

1. 本报告只对送检的来样负责。
2. 检验报告上的检验结果和检验单位名称，未经同意不得用于广告、评优及商业宣传。
3. 对本报告有异议，请于收到报告之日起十五日内以书面方式提出，逾期不予受理。
4. 对纸质检验报告涂改、增删，或未加盖检验单位印章的复印件均无效。

上海翼和应用生物技术有限公司 联系地址：上海市漕河泾开发区桂平路 471 号桂果园 8 号楼 4F 室
网址：http://www.biowing.com.cn/邮箱：biowing@vip.163.com 服务热线：021-33559491

样 品 信 息

样品编号：

客户样本编号	公司编号
DU145	20190902-01

样品数量：1

样品性状：细胞系

检测项目：STR

送检单位：赛百慷

检测方法：用 Axygen 的基因组抽提试剂盒提取 DNA，采用 20- STR 扩增方案扩增，在 ABI 3730XL 型遗传分析仪上对 STR 位点和性别基因 Amelogenin 进行检测。

检 测 结 果

(一) 检验基本情况

	多等位基因	匹配细胞系	细胞库	EV 值	匹配说明
20190902-01	有	DU 145	DSMZ	0.88	基本匹配

样本基因型检验结果

- 多等位基因指三等位及以上基因现象。
- 本次检测各细胞分型结果良好。

(二) 各样本描述

- 20190902-01：该株细胞 DNA 分型在细胞系检索中找到**基本匹配**的细胞系，DSMZ 数据库显示细胞名为 **DU 145**，细胞号对应 **HTB-81** 。本次检测在该细胞系中**发现多等位基因**。

  备注：待测细胞系与收录于 ATCC, DSMZ, JCRB 和 RIKEN 数据库的细胞系 STR 数据进行比对，未收录于以上细胞库的细胞系将无法匹配。

（三）样本分型结果

细胞 20190902-01 的 STR 位点和 Amelogenin 位点的基因分型结果						
Loci	送检细胞 STR 信息			细胞库细胞 STR 信息		
	送检细胞名：DU145			细胞库细胞名：DU 145		
	Allele1	Allele2	Allele3	Allele1	Allele2	Allele3
D5S818	9	9		10	13	
D13S317	12	13	14	12	13	14
D7S820	7	10	11	7	10	11
D16S539	11	13		11	13	
VWA	17	18		17	18	19
TH01	7	7		7	7	
AMEL	X	Y		X	Y	
TPOX	11	11		11	11	
CSF1PO	10	11		10	11	
D12S391	18	21				
FGA	22	22				
D2S1338	16	17				
D21S11	31	33				
D18S51	12	12				
D8S1179	13	14				
D3S1358	16	16				
D6S1043	10	11				
PENTAE	12	14				
D19S433	13	13				
PENTAD	8.2	12.2				

上海翼和应用生物技术有限公司 联系地址：上海市漕河泾开发区桂平路 471 号桂果园 8 号楼 4F 室
网址：http://www.biowing.com.cn/邮箱：biowing@vip.163.com 服务热线：021-33559491

其他说明

(一) 分型方案及位点分布

	方案 1	方案 2	方案 3	方案 4
1	TH01	TPOX	D3S1358	AMEL
2	D12S391	VWA	D13S317	D5S818
3	D7S820	D8S1179	D6S1043	D2S1338
4	CSF1PO	PENTAD	D16S539	D21S11
5	FGA		D19S433	D18S51
6	PENTAE			

实验方案及位点

(二) STR 数据库比对

本公司采用 DSMZ tools 进行细胞系比对，其中包含来自于 ATCC, DSMZ, JCRB 和 RIKEN 数据库的 2455 个细胞系 STR 数据。如果待检测细胞未收录于以上细胞库或这是自行建立的新细胞系将无法进行比对，用户需根据细胞分型结果自行与其他数据库进行比对。

(三) 文献引用参考

1 . Authentication testing of HEK 293T and HeLa cell lines have been performed by Shanghai Biowing Applied Biotechnology Co.,Ltd via STR profiling. STR profiles match the standards recommended for HEK 293T and HeLa cell lines authentication

2 . AGS, NCI-N87, HGC-27 and HEK293 were STR-authenticated on Dec. 8, 2015 by Shanghai Biowing Applied Biotechnology Co. LTD, Shanghai, China

主要实验人员：张佳男

复核人: 龚礼瑾

负责人：陈轶群

签发日期：2019 年 09 月 16 日

上海翼和应用生物技术有限公司 联系地址：上海市漕河泾开发区桂平路 471 号桂果园 8 号楼 4F 室
网址：http://www.biowing.com.cn/邮箱：biowing@vip.163.com 服务热线：021-33559491

Applied Biosystems

GeneMapper 4.0

Cell Line Authentication 1129 1201

Sample File	Sample Name	Panel	OS	SQ
35 C05.fsa	DU145	STR Profile 1-human		

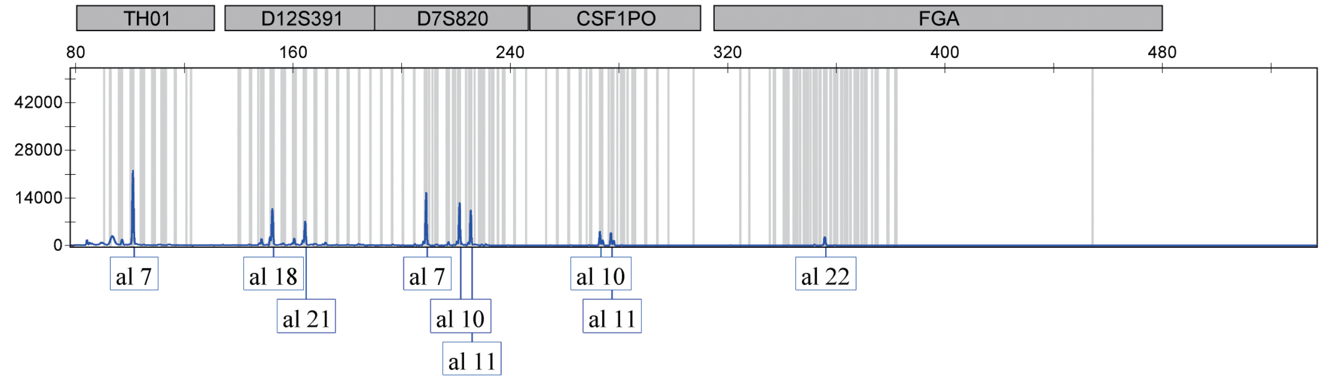

Sample File	Sample Name	Panel	OS	SQ
35 C05.fsa	DU145	STR Profile 1-human		

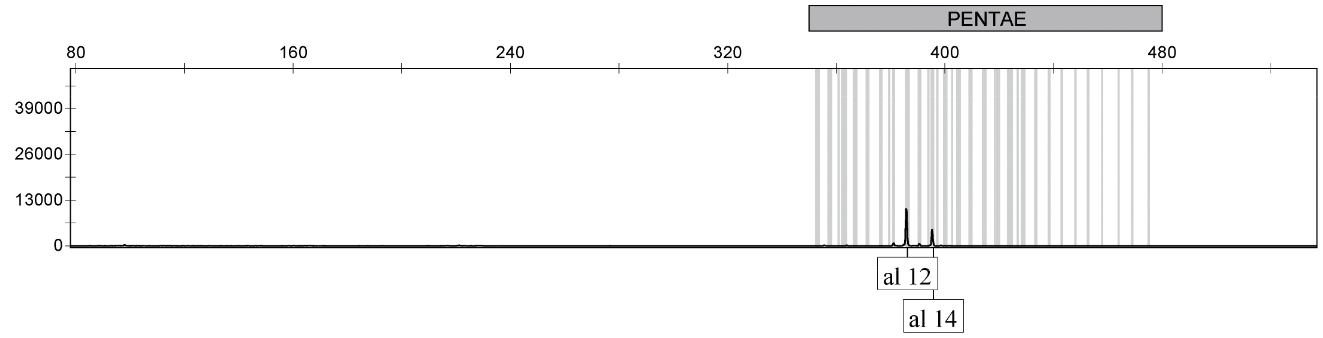

Sample File	Sample Name	Panel	OS	SQ
75 C10.fsa	DU145	STR Profile 2-human		

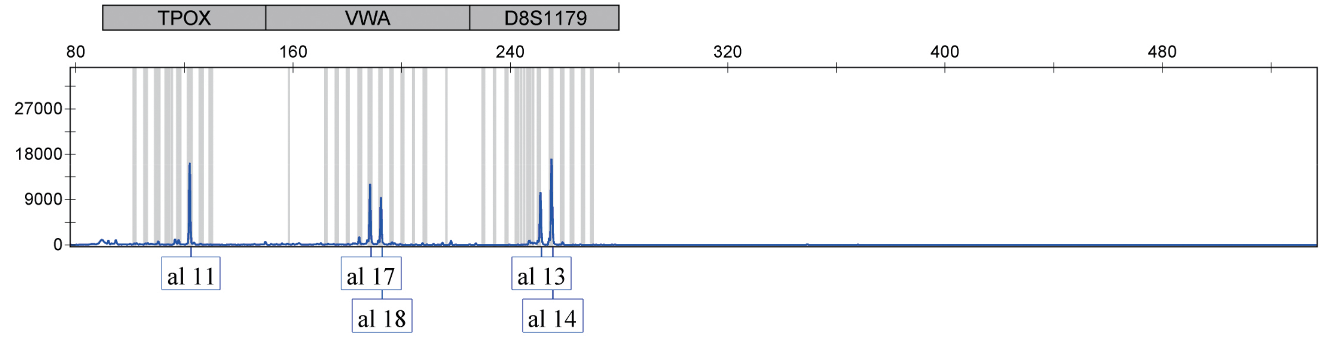

Applied Biosystems

GeneMapper 4.0

Cell Line Authentication 1129 1201

Sample File	Sample Name	Panel	OS	SQ
75 C10.fsa	DU145	STR Profile 2-human		

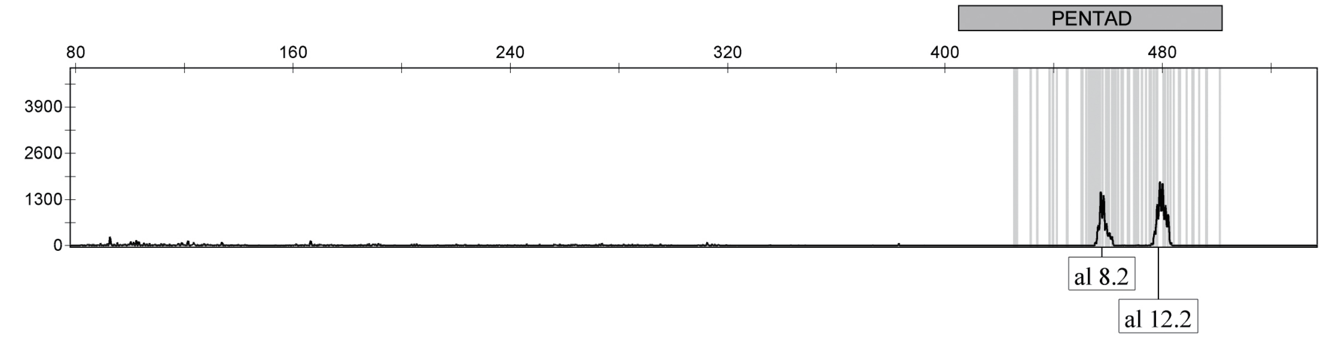

Sample File	Sample Name	Panel	OS	SQ
03 C01.fsa	DU145	STR Profile 3-human		

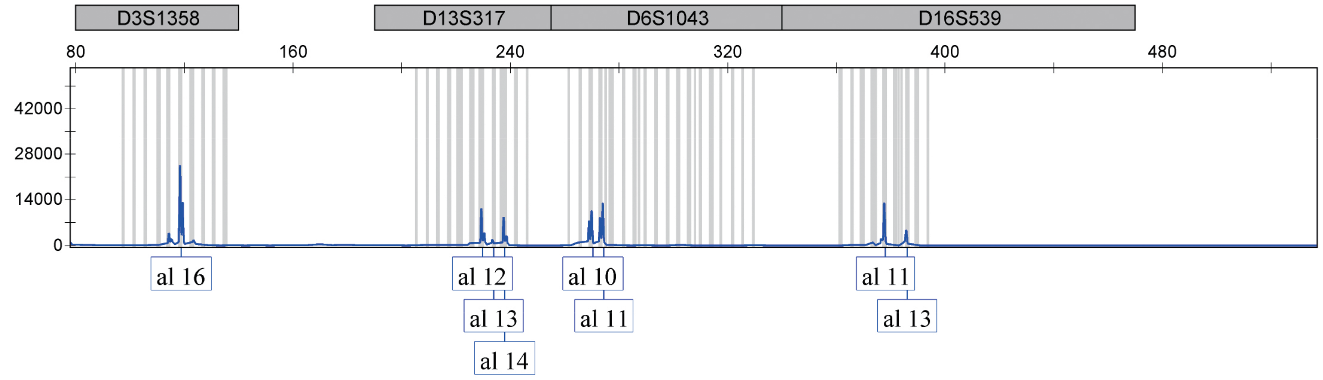

Sample File	Sample Name	Panel	OS	SQ
03 C01.fsa	DU145	STR Profile 3-human		

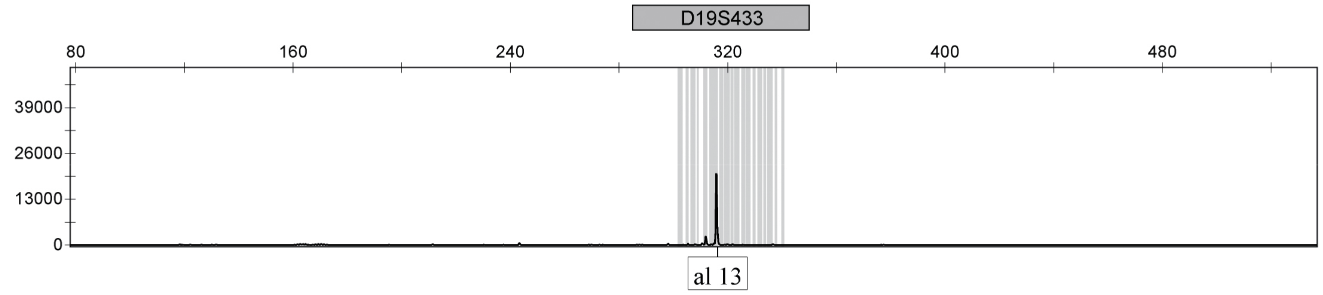

Applied Biosystems

GeneMapper 4.0

Cell Line Authentication 1129 1201

Sample File	Sample Name	Panel	OS	SQ
35_C05.fsa	DU145	STR Profile 4-human		

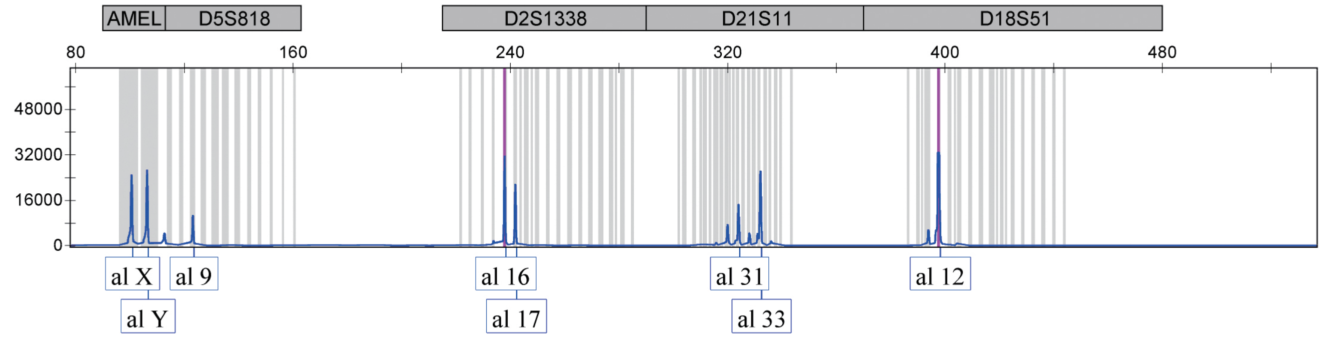

35_C05.fsa	DU145	STR Profile 4-human		

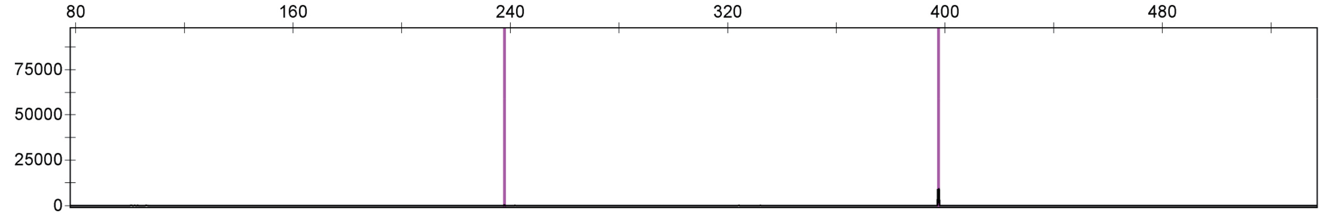

Sample File	Sample Name	Panel	SQO	OS	SQ
40_H05_CellLineAuthentication-1-0903.fsa	+	21Plex_STR_Panel_v0.4			

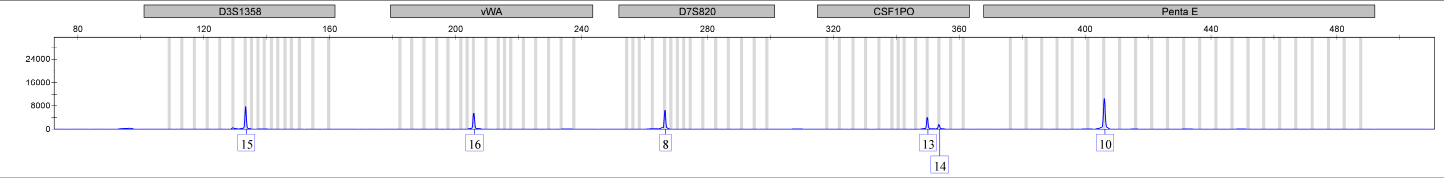

Sample File	Sample Name	Panel	SQO	OS	SQ
40_H05_CellLineAuthentication-1-0903.fsa	+	21Plex_STR_Panel_v0.4			

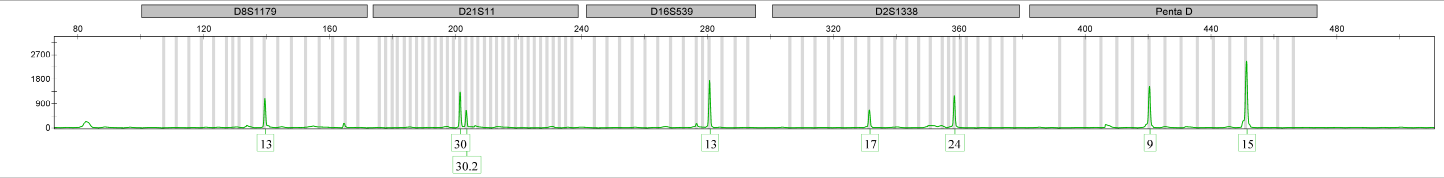

Sample File	Sample Name	Panel	SQO	OS	SQ
40_H05_CellLineAuthentication-1-0903.fsa	+	21Plex_STR_Panel_v0.4			

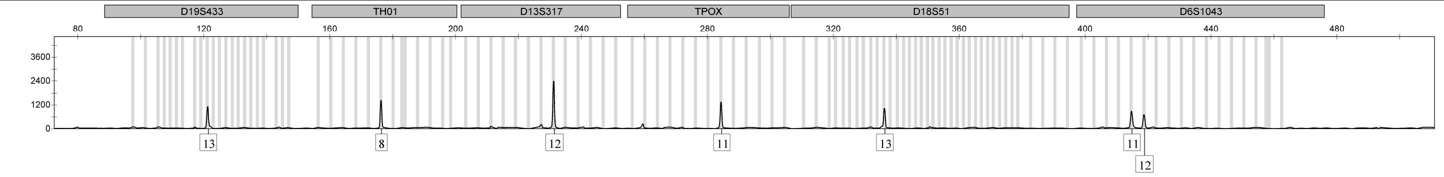

Sample File	Sample Name	Panel	SQO	OS	SQ
40_H05_CellLineAuthentication-1-0903.fsa	+	21Plex_STR_Panel_v0.4			

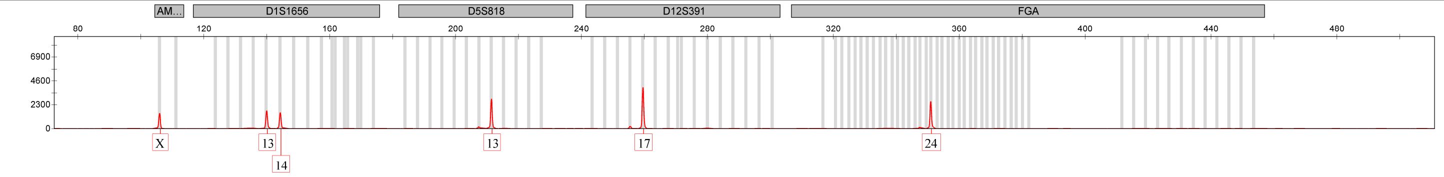

Supplement: Supplementary file 7 — Additional file 7. [file 13046_2021_2138_MOESM7_ESM.zip › DU145 STR.pdf]
