# Supplementary material for: Hsa_circ_0004296 inhibits metastasis of prostate cancer by interacting with EIF4A3 to prevent nuclear export of ETS1 mRNA
Source: J Exp Clin Cancer Res. 2021 Oct 25;40:336. doi: 10.1186/s13046-021-02138-8 (PMC8543852; doi:10.1186/s13046-021-02138-8)

# 细胞遗传质量鉴定检测

# Cell Line Authentication Service

## STR 基因型检测报告

**送检单位：赛百慷**

**检品名称：**细胞系

**委托单位：**上海翼和应用生物技术有限公司

**报告日期：**2019-09-25


上海翼和应用生物技术有限公司 联系地址：上海市漕河泾开发区桂平路 471 号桂果园 8 号楼 4F 室
网址：http://www.biowing.com.cn/ 邮箱：biowing@vip.163.com 服务热线：021-33559491

# 报 告 说 明

1. 本报告只对送检的来样负责。
2. 检验报告上的检验结果和检验单位名称，未经同意不得用于广告、评优及商业宣传。
3. 对本报告有异议，请于收到报告之日起十五日内以书面方式提出，逾期不予受理。
4. 对纸质检验报告涂改、增删，或未加盖检验单位印章的复印件均无效。


上海翼和应用生物技术有限公司 联系地址：上海市漕河泾开发区桂平路 471 号桂果园 8 号楼 4F 室
网址：http://www.biowing.com.cn/ 邮箱：biowing@vip.163.com 服务热线：021-33559491

# 样 品 信 息

**样品编号：**

| 客户样本编号 | 公司编号 |
| --- | --- |
| PC-3 | 20190916-05 |

**样品数量：**1

**样品性状：**细胞系

**检测项目：**STR

**送检单位：**赛百慷

**检测方法：**用 Axygen 的基因组抽提试剂盒提取 DNA，采用 20- STR 扩增方案扩增，在 ABI 3730XL 型遗传分析仪上对 STR 位点和性别基因 Amelogenin 进行检测。


上海翼和应用生物技术有限公司 联系地址：上海市漕河泾开发区桂平路 471 号桂果园 8 号楼 4F 室
网址：http://www.biowing.com.cn/ 邮箱：biowing@vip.163.com 服务热线：021-33559491

# 检 测 结 果

## (一) 检验基本情况

| | 多等位基因 | 匹配细胞系 | 细胞库 | EV 值 | 匹配说明 |
|---|---|---|---|---|---|
| 20190916-05 | 无 | PC-3 | DSMZ | 0.94 | 基本匹配 |

样本基因型检验结果

- 多等位基因指三等位及以上基因现象。
- 本次检测各细胞分型结果良好。

## (二) 各样本描述

- 20190916-05：该株细胞 DNA 分型在细胞系检索中找到**基本匹配**的细胞系，DSMZ 数据库显示细胞名为 **PC-3**，细胞号对应 **465**。本次检测在该细胞系中**没有发现多等位基因**。

  **备注：**待测细胞系与收录于 ATCC, DSMZ, JCRB 和 RIKEN 数据库的细胞系 STR 数据进行比对，未收录于以上细胞库的细胞系将无法匹配。


上海翼和应用生物技术有限公司 联系地址：上海市漕河泾开发区桂平路 471 号桂果园 8 号楼 4F 室
网址：http://www.biowing.com.cn/ 邮箱：biowing@vip.163.com 服务热线：021-33559491

**（三）样本分型结果**

| 细胞 20190916-05 的 STR 位点和 Amelogenin 位点的基因分型结果 | | | | | | |
|---|---|---|---|---|---|---|
| Loci | 送检细胞 STR 信息 | | | 细胞库细胞 STR 信息 | | |
| | 送检细胞名：PC-3 | | | 细胞库细胞名：PC-3 | | |
| | Allele1 | Allele2 | Allele3 | Allele1 | Allele2 | Allele3 |
| D5S818 | 13 | 13 | | 13 | 13 | |
| D13S317 | 11 | 11 | | 11 | 11 | |
| D7S820 | 8 | 11 | | 8 | 11 | |
| D16S539 | 11 | 11 | | 11 | 11 | |
| VWA | 17 | 17 | | 17 | 17 | |
| TH01 | 6 | 7 | | 6 | 7 | |
| AMEL | X | X | | X | X | |
| TPOX | 8 | 8 | | 8 | 9 | |
| CSF1PO | 11 | 11 | | 11 | 11 | |
| D12S391 | 21 | 21 | | | | |
| FGA | 25 | 25 | | | | |
| D2S1338 | 18 | 20 | | | | |
| D21S11 | 29 | 31.2 | | | | |
| D18S51 | 14 | 15 | | | | |
| D8S1179 | 13 | 13 | | | | |
| D3S1358 | 16 | 16 | | | | |
| D6S1043 | 14 | 18 | | | | |
| PENTAE | 10 | 17 | | | | |
| D19S433 | 14 | 14 | | | | |
| PENTAD | 8.1 | 8.1 | | | | |


上海翼和应用生物技术有限公司　联系地址：上海市漕河泾开发区桂平路 471 号桂果园 8 号楼 4F 室
网址：http://www.biowing.com.cn/　邮箱：biowing@vip.163.com　服务热线：021-33559491

# 其他说明

## (一)　分型方案及位点分布

| | 方案 1 | 方案 2 | 方案 3 | 方案 4 |
|---|---|---|---|---|
| 1 | TH01 | TPOX | D3S1358 | AMEL |
| 2 | D12S391 | VWA | D13S317 | D5S818 |
| 3 | D7S820 | D8S1179 | D6S1043 | D2S1338 |
| 4 | CSF1PO | PENTAD | D16S539 | D21S11 |
| 5 | FGA | | D19S433 | D18S51 |
| 6 | PENTAE | | | |

实验方案及位点

## (二)　STR 数据库比对

本公司采用 DSMZ tools 进行细胞系比对，其中包含来自于 ATCC, DSMZ, JCRB 和 RIKEN 数据库的 2455 个细胞系 STR 数据。如果待检测细胞未收录于以上细胞库或这是自行建立的新细胞系将无法进行比对，用户需根据细胞分型结果自行与其他数据库进行比对。

## (三)　文献引用参考

1．Authentication testing of HEK 293T and HeLa cell lines have been performed by Shanghai Biowing Applied Biotechnology Co.,Ltd via STR profiling. STR profiles match the standards re commended for HEK 293T and HeLa cell lines authentication

2．AGS, NCI-N87, HGC-27 and HEK293 were STR-authenticated on Dec. 8, 2015 by Shanghai Biowing Applied Biotechnology Co. LTD, Shanghai, China

**主要实验人员：**张佳男

**复核人:** 龚礼瑾

**负责人：**陈轶群

**签发日期：**2019 年 09 月 25 日


上海翼和应用生物技术有限公司　联系地址：上海市漕河泾开发区桂平路 471 号桂果园 8 号楼 4F 室
网址：http://www.biowing.com.cn/　邮箱：biowing@vip.163.com　服务热线：021-33559491

Applied Biosystems
GeneMapper 4.0

CellLineAuthentication-0228

| Sample File | Sample Name | Panel | SQI | OS | SQ |
|---|---|---|---|---|---|
| 18_B03_CellLineAuthentication-1-0308.fsa | PC-3m | STR Profile 1-human | | | |

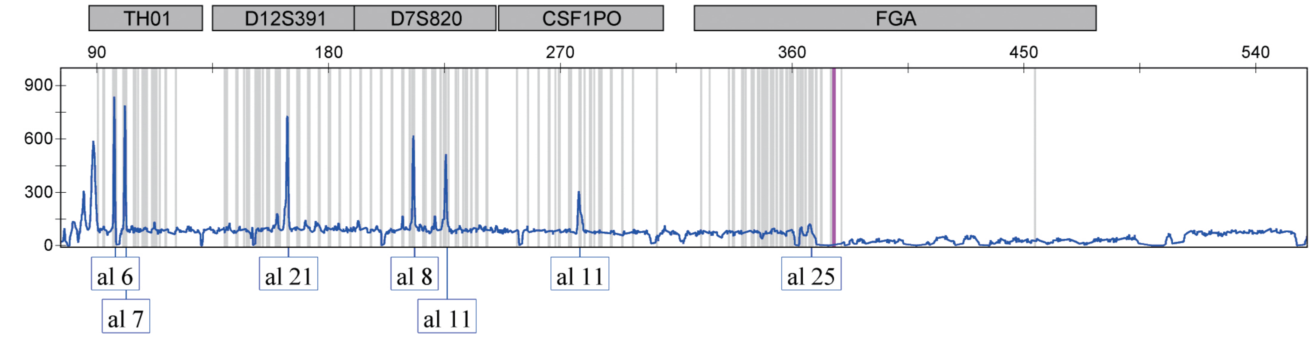


| Sample File | Sample Name | Panel | SQI | OS | SQ |
|---|---|---|---|---|---|
| 18_B03_CellLineAuthentication-1-0308.fsa | PC-3m | STR Profile 1-human | | | |

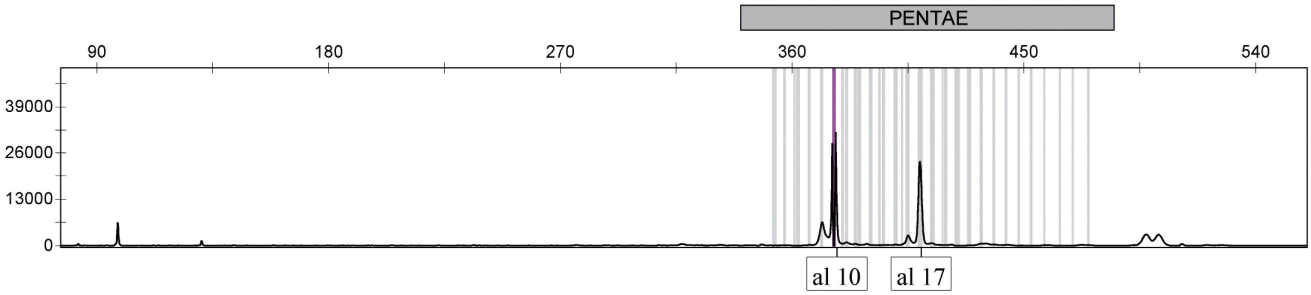


| Sample File | Sample Name | Panel | SQI | OS | SQ |
|---|---|---|---|---|---|
| 42_B06_CellLineAuthentication-1-0308.fsa | PC-3m | STR Profile 2-human | | | |

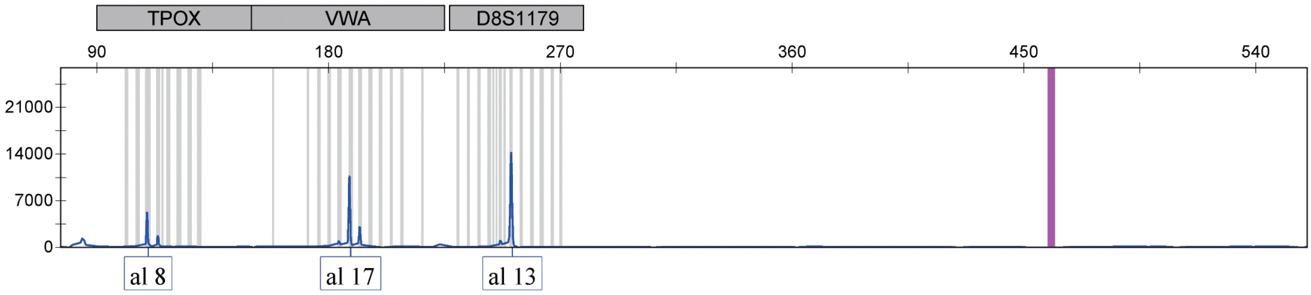


| Sample File | Sample Name | Panel | SQI | OS | SQ |
|---|---|---|---|---|---|
| 42_B06_CellLineAuthentication-1-0308.fsa | PC-3m | STR Profile 2-human | | | |

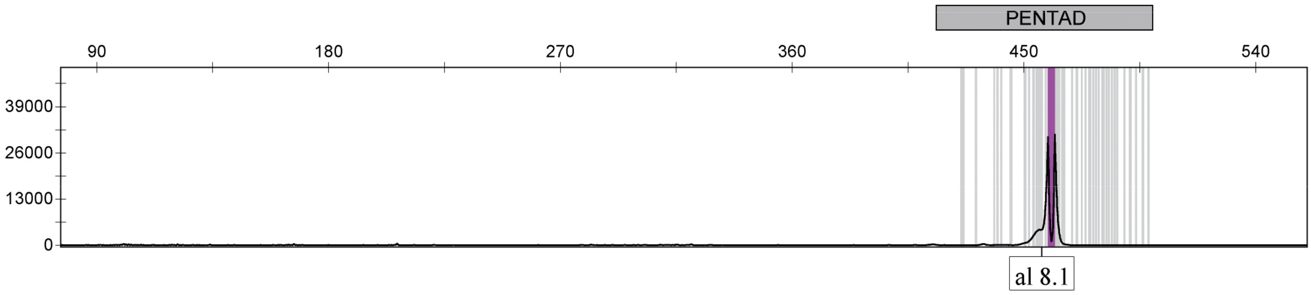

GeneMapper 4.0

CellLineAuthentication-0228

| Sample File | Sample Name | Panel | SQI | OS | SQ |
|---|---|---|---|---|---|
| 66_B09_CellLineAuthentication-1-0308.fsa | PC-3m | STR Profile 3-human | | | |

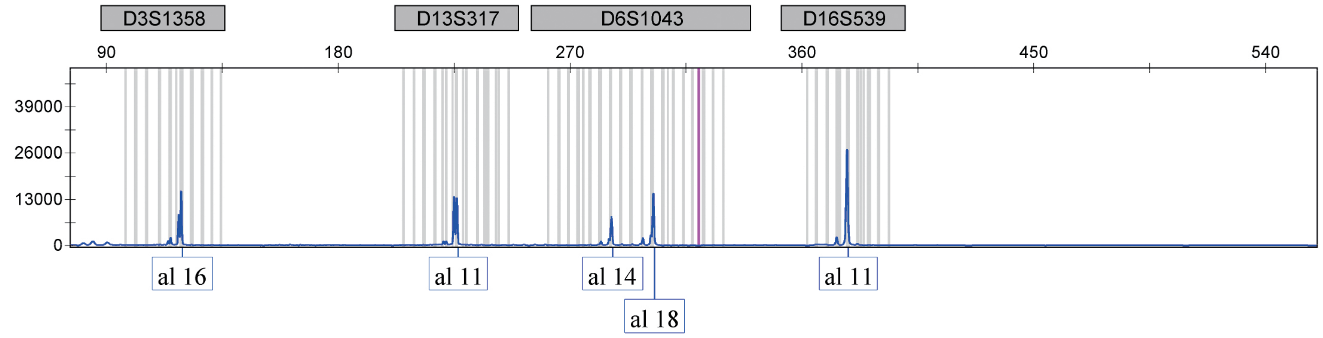


| Sample File | Sample Name | Panel | SQI | OS | SQ |
|---|---|---|---|---|---|
| 66_B09_CellLineAuthentication-1-0308.fsa | PC-3m | STR Profile 3-human | | | |

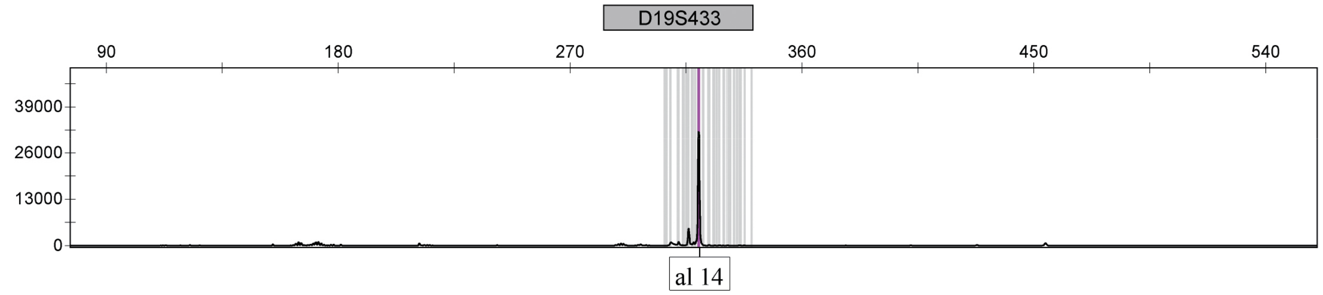


| Sample File | Sample Name | Panel | SQI | OS | SQ |
|---|---|---|---|---|---|
| 90_B12_CellLineAuthentication-1-0308.fsa | PC-3m | STR Profile 4-human | | | |

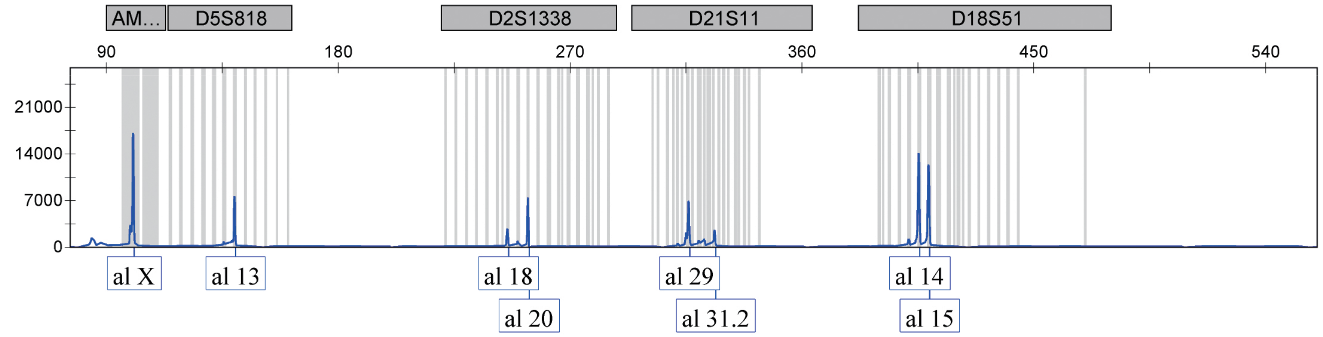


| Sample File | Sample Name | Panel | SQI | OS | SQ |
|---|---|---|---|---|---|
| 90_B12_CellLineAuthentication-1-0308.fsa | PC-3m | STR Profile 4-human | | | |

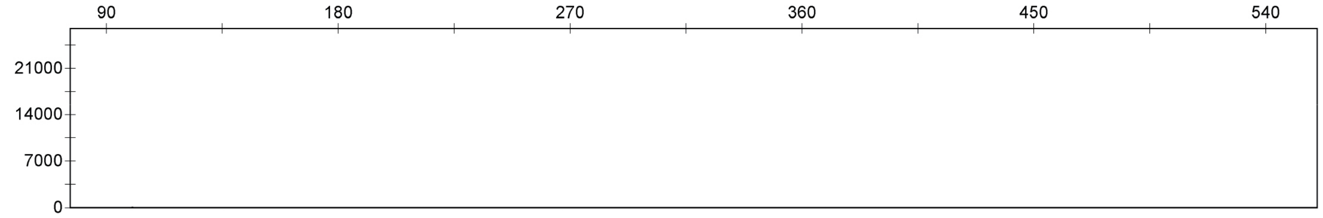

Applied Biosystems

GeneMapper ID v3.2

0912

| Sample File | Sample Name | Panel | OS | SQ |
|---|---|---|---|---|
| 06_F01_CellLineAuthentication-1-0916.fsa | + | 21Plex_STR_Panel_v1.1 | | |

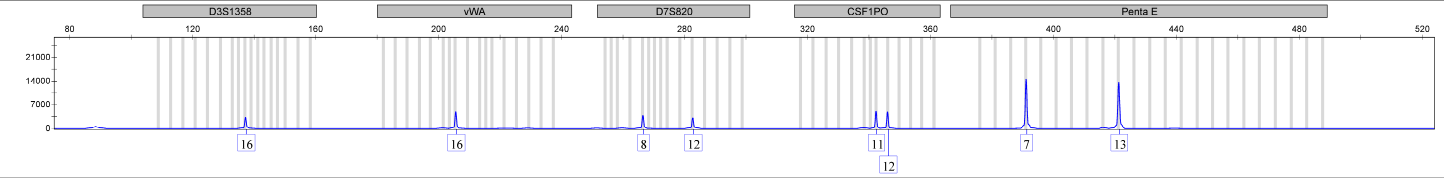


| | | | | |
|---|---|---|---|---|
| 06_F01_CellLineAuthentication-1-0916.fsa | + | 21Plex_STR_Panel_v1.1 | | |

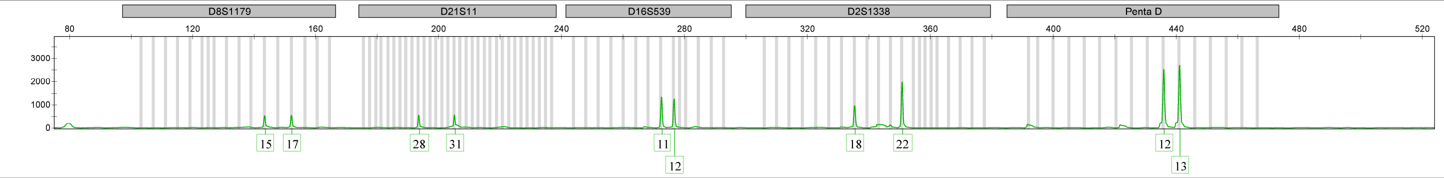


| | | | | |
|---|---|---|---|---|
| 06_F01_CellLineAuthentication-1-0916.fsa | + | 21Plex_STR_Panel_v1.1 | | |

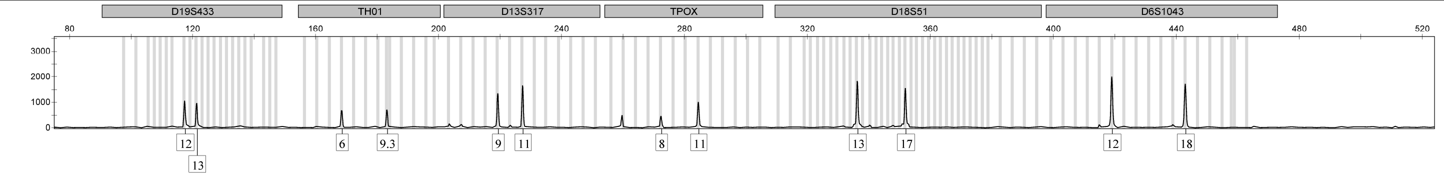


| | | | | |
|---|---|---|---|---|
| 06_F01_CellLineAuthentication-1-0916.fsa | + | 21Plex_STR_Panel_v1.1 | | |

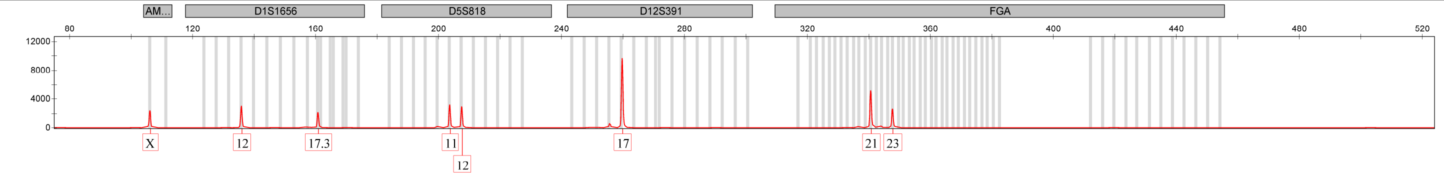

Supplement: Supplementary file 7 — Additional file 7. [file 13046_2021_2138_MOESM7_ESM.zip › PC3 STR.pdf]
