# Supplementary material for: Hsa_circ_0004296 inhibits metastasis of prostate cancer by interacting with EIF4A3 to prevent nuclear export of ETS1 mRNA
Source: J Exp Clin Cancer Res. 2021 Oct 25;40:336. doi: 10.1186/s13046-021-02138-8 (PMC8543852; doi:10.1186/s13046-021-02138-8)
Supplement: Supplementary file 7 — Additional file 7. [file 13046_2021_2138_MOESM7_ESM.zip › RWPE-1 STR.pdf]

细胞遗传质量鉴定检验报告

检品名称：细胞系

检验类型：STR 基因型检验

样品编号：

表 1 样本编号

客户样本编号	公司编号
250	20170410-16

样品数量：1

样品性状：细胞系

检测项目：STR

检测方法：用 Axygen 的基因组抽提试剂盒提取 DNA，采用 20- STR 扩增方案扩增，在 ABI 3730XL 型遗传分析仪上对 STR 位点和性别基因 Amelogenin 进行检测。

检验结果：

(一)检验基本情况

表 2：样本基因型检验结果

	多等位基因	匹配细胞系	细胞库	EV 值	匹配说明
20170410-16	有	RWPE-1	DSMZ	0.92	基本匹配

- 多等位基因指三等位及以上基因现象。
- 本次检测各细胞分型结果良好。

(二)各样本描述

- 20170410-16：该株细胞 DNA 分型在细胞系检索中找到基本匹配的细胞系，DSMZ 数据库显示细胞名为 RWPE-1，细胞号对应 CRL-11609。本次检测在该细胞系中发现多等位基因。

(三)样本分型结果

表 3：细胞 20170410-16 的 STR 位点和 Amelogenin 位点的基因分型结果

Marker	样本				细胞库信息		
	Allele1	Allele2	Allele3	Allele4	Allele1	Allele2	Allele3
D5S818	12	15			12	15	
D13S317	8	9.3	14		8	14	
D7S820	10	11			10	11	
D16S539	9	11			9	11	
VWA	14	18			14	18	
TH01	8	9.3			8	9.3	
AMEL	X	X			X	Y	
TPOX	8	11			8	11	
CSF1PO	13	13			13	13	
D12S391	20	23					
FGA	24	25					
D2S1338	17	20					
D21S11	29	30	31				
D18S51	16	16					
D8S1179	10	14					
D3S1358	16	16					
D6S1043	18	19					
PENTAE	5	12					
D19S433	13	13					
PENTAD	10	13					

其他说明：

(一)分型方案及位点分布：

附表：实验方案及位点

	方案 1	方案 2	方案 3	方案 4
1	TH01	TPOX	D3S1358	AMEL
2	D12S391	VWA	D13S317	D5S818
3	D7S820	D8S1179	D6S1043	D2S1338
4	CSF1PO	PENTAD	D16S539	D21S11
5	FGA		D19S433	D18S51
6	PENTAE			

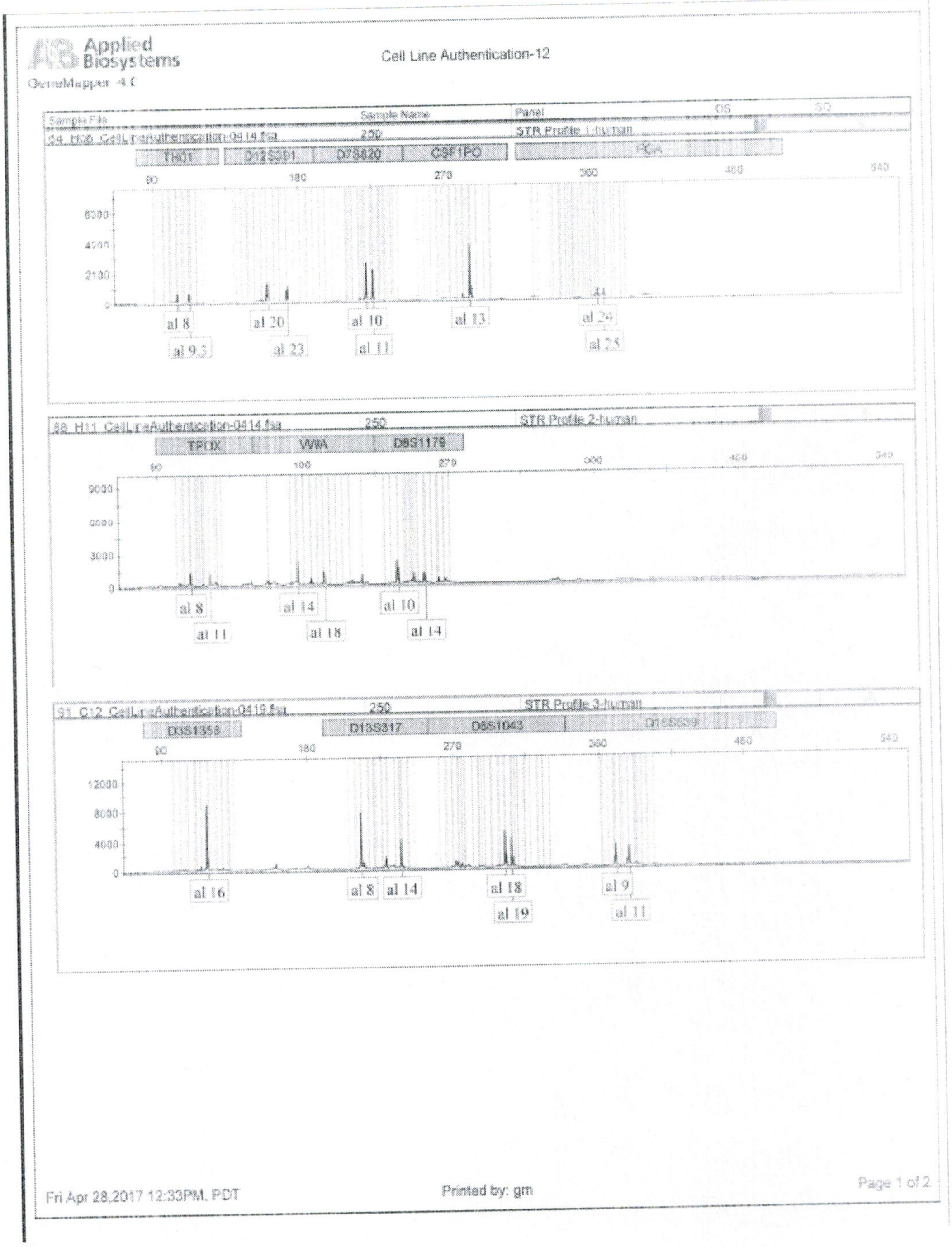
Applied Biosystems
GeneMapper 4.0
Cell Line Authentication-12
Sample File
Sample Name
Panel
250
STR Profile 1-human
TH01
D21S11
D7S820
CSF1PO
FGA
al 8
al 9.3
al 20
al 23
al 10
al 11
al 13
al 24
al 25
250
STR Profile 2-human
TPOX
VWA
D8S1179
al 8
al 11
al 14
al 18
al 10
al 14
250
STR Profile 3-human
D3S1358
D13S317
D5S1043
al 16
al 8
al 14
al 18
al 19
al 9
al 11
Fri Apr 28,2017 12:33PM, PDT
Printed by: gm
Page 1 of 2

Applied Biosystems

Cell Line Authentication-12

GeneMapper 4.0

Sample File	Sample Name	Panel	QS	SQ
24_H03_CellLineAuthentication-04 l4.fsa	250	STR Profile 4-Human		

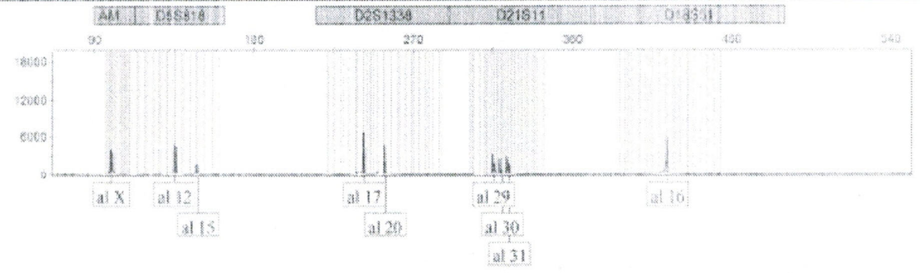

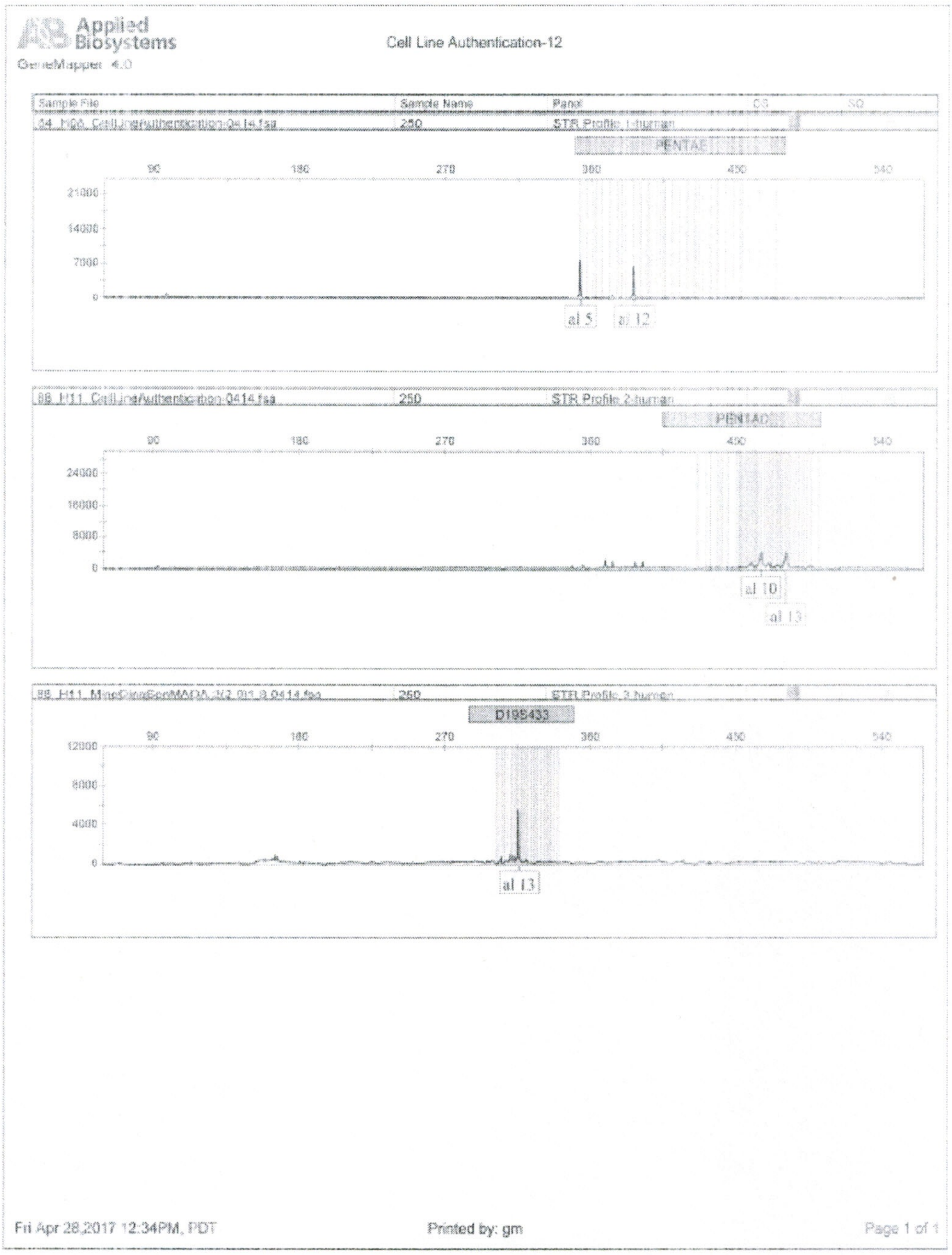

签发日期：
2017 年 05 月
